# Multi-Omics Profiling Identifies Immune–Metabolic Signatures and Gut Microbial Biomarkers in a Murine Model of *Mycoplasma pneumoniae* Pneumonia

**DOI:** 10.3390/biology15171469

**Published:** 2026-08-31

**Authors:** Yun Li, Changbai Hu, Yunwei Liu, Jiawen Ye, Tong Zhou, Jiaoxu Shi, Lin Xiao, Lingyun Yang, Dongxuan Li, Lianyu Wang, Xiaoying Wen, Yongyao Yu, Jinghua Yang, Xiaolan Xiao

**Affiliations:** 1 Department of Pediatrics, The Second Affiliated Hospital, Guangzhou University of Chinese Medicine, Guangzhou 510030, China; 2 Hubei-Hongshan Laboratory, Huazhong Agricultural University, Wuhan 430070, China

**Keywords:** *Mycoplasma pneumoniae* pneumonia, multi-omics, immune–metabolic crosstalk, gut–lung axis, biomarkers

## Abstract

*Mycoplasma pneumoniae* pneumonia (MPP) is driven by excessive host immune responses and metabolic dysregulation, yet its systemic pathogenesis remains poorly understood. Here, we established a murine MPP via MP (1 × 10^8^ CCU/mL), which faithfully recapitulates clinical features, including interstitial pneumonia, elevated pro-inflammatory cytokines (IL-1β, IL-6, TNF-α), and increased alveolar macrophage abundance. Integrating lung transcriptomics and metabolomics, we found that MP infection activates pro-inflammatory cytokines (e.g., cytokine–cytokine receptor interaction, Th17 differentiation, NF-κB signaling) while disrupting fatty acid metabolism. Correlative analyses revealed that downregulated 3′-AMP and CMP-Neu5Ac correlated with elevated pro-inflammatory *IL-18*/*IL-33*, whereas upregulated 4-imidazoleacrylic acid correlated with reduced anti-inflammatory *IL-12*A base on transcriptomic data, suggesting that metabolic reprogramming may exacerbate pulmonary inflammation. Furthermore, 16S rRNA sequencing and fecal metabolomics revealed marked microbiota dysbiosis, enrichment of *Marvinbryantia*, and depletion of beneficial taxa, alongside altered fecal metabolites, with correlations linking gut microbial shifts and metabolite changes to lung transcript-level inflammatory cytokines, supporting a gut–lung axis involvement in MPP. Collectively, our multi-omics dissection provides a systems-level view of immune–metabolic crosstalk in MP infection, offering mechanistic insights and potential biomarkers for improved diagnosis and therapeutic targeting.

## Simple Summary

*Mycoplasma pneumoniae* pneumonia (MPP) is a common respiratory infection with poorly understood systemic pathogenesis. Using a murine model, we integrated lung transcriptomics, lung metabolomics, fecal 16S rRNA sequencing, and fecal metabolomics to systematically characterize host responses to *Mycoplasma pneumoniae* (MP) infection. We found that MP infection activates pulmonary inflammatory pathways (NF-κB, Th17) while disrupting fatty acid metabolism. Notably, three pulmonary metabolites (3′-AMP, CMP-Neu5Ac, and 4-imidazoleacrylic acid) showed significant correlations with key transcript-level inflammatory cytokines (*IL-18*, *IL-33*, *IL-12A*), implicating metabolic reprogramming in pulmonary inflammation. Concurrent fecal 16S rRNA and metabolomic analyses showed marked gut dysbiosis (*Marvinbryantia* enrichment) and altered metabolites linked to lung transcript-level cytokines, providing evidence for gut–lung axis involvement. Our multi-omics dissection offers a systems-level view of immune–metabolic crosstalk in MPP and identifies candidate biomarkers from both the pulmonary and intestinal compartments, with potential applications in diagnosis and therapeutic targeting.

## 1. Introduction

*Mycoplasma pneumoniae* pneumonia (MPP), caused by *Mycoplasma pneumoniae* (MP), accounts for 10–40% of community-acquired pneumonia (CAP) cases, with clinical severity ranging from mild self-limiting illness to life-threatening necrotizing pneumonia or respiratory failure [1]. The pathogen exhibits epidemic cycles every 3–7 years with considerable regional variation [1,2], and recent epidemiological reports have documented a marked resurgence of MP infections [3,4,5,6]. In clinical practice, treatment is increasingly hampered by sharply rising antimicrobial resistance rates and associated side effects [7,8]. Notably, targeting the NLRP3 inflammasome has been proposed as a potential strategy [9,10] and adjunctive corticosteroid therapy improves outcomes in severe cases [11,12], suggesting that lung injury is primarily driven by an exaggerated host immune response rather than direct pathogen cytotoxicity. However, the underlying immunopathogenesis remains largely elusive, and a systematic understanding of pulmonary immune homeostasis is critically needed to improve therapeutic outcomes.

The immunopathogenesis of MPP is sustained by a self-perpetuating cycle orchestrated by host immune dysfunction and microbial immune evasion [13]. In the innate compartment, macrophages exhibit a critical polarization imbalance—impaired M1-mediated TLRs/MyD88/NF-kB signaling [14,15], alongside expanded immunosuppressive M2-like populations expressing PD-L1 and IDO1 that suppress protective T-cell responses [16]. In adaptive immunity, disrupted T-cell homeostasis is central to disease progression: reduced CD4^+^/CD8^+^ ratio [16,17], a diminished Th1/Th2 ratio favoring Th2 dominance [17,18,19], and an impaired Treg/Th17 balance that unleashes hyperinflammation, collectively impairing pathogen clearance and promoting tissue injury [20,21,22]. Despite these mechanistic clues, most findings derive from isolated clinical observations, and a systems-level characterization of pulmonary immune perturbations in a controlled experimental setting is urgently required.

Concurrent with immune activation, acute MP infection triggers profound metabolic reprogramming within the lung microenvironment. Metabolomic studies of MPP patients have revealed significant perturbations in lipid, tryptophan, and glycerophospholipid metabolism in both serum and bronchoalveolar lavage fluid (BALF) [23,24], with elevated short-chain fatty acids (SCFAs), such as acetic and isobutyric acid, as potential circulating biomarkers [24]. Transcriptomic profiling has further identified upregulated gene modules related to mononuclear cell proliferation and signaling (e.g., RLTPR, CARD11, RASAL3) in pulmonary samples from MPP children [25,26]. In murine models, metabolomic analysis of serum following MP infection revealed significant alterations in steroid hormone metabolites, including cortisol, and disruption of the steroid hormone synthesis pathway [27]. In addition, transcriptomic studies in mouse models have explored pulmonary transcriptional changes after MP infection and found that ZBP1 mRNA expression was markedly upregulated, suggesting that MP infection promotes macrophage activation and alleviates inflammation and barrier damage [28].

Beyond their role as passive biomarkers, metabolites are active participants in immune regulation. For instance, MP infection has been shown to activate STAT3, driving citrate accumulation in airway epithelial cells, which in turn modulates the balance between inflammatory responses and pathogen clearance through adenosine triphosphate-citrate lyase (ACLY)-mediated metabolic reprogramming [29]. From a broader perspective, metabolites such as succinate, lactate, and kynurenine have been identified as signaling molecules that directly modulate immune responses and inflammatory states [30,31].

Notably, MP infection not only affects lung tissue but may also influence the intestinal microecology. The characterization of the gut–lung axis in the context of MP infection remains largely unexplored. Most current multi-omics studies are primarily based on single-omics analyses of clinical specimens, and there are few systematic studies that simultaneously integrate both immunological and metabolic dimensions in controlled animal models [23,24,25]. Therefore, this study adopted a multi-omics integration strategy to systematically characterize the immunometabolism and microbial changes in the lungs and intestines of mice following MP infection, with the aim of providing valuable hypotheses regarding the mechanisms underlying the lung–gut axis.

Beyond the lung, the gut–lung axis has emerged as a critical modulator of respiratory infections. Microbiome investigations have shown that MPP patients exhibit not only decreased pulmonary microbial α-diversity with increased MP abundance [32], but also significant gut dysbiosis, characterized by reduced beneficial genera such as *Ruminococcus flavefaciens*, *Clostridium butyricum*, *Lactobacillus*, and *Bifidobacterium* [33], alongside diminished *Roseburia* and a predominance of *Prevotella* and *Enterococcus* in the MPP group [34]. Notably, *Bifidobacterium* or *Lactobacillus* can induce regulatory T-cell differentiation and suppress inflammation through SCFAs, mitigating tissue damage [35,36,37]. Therefore, the loss of these bacteria may contribute to inadequate anti-inflammatory regulation, ultimately favouring the development of severe inflammatory responses during MP infection. Given that gut microbiota and their products (e.g., SCFA) are pivotal mediators of the gut–lung axis [38,39] findings suggest that intestinal microbial and metabolic perturbations may serve as non-invasive diagnostic biomarkers for MPP. Nevertheless, current evidence is predominantly cross-sectional and descriptive, lacking integrated multi-omics data and causal validation, thus leaving the functional contribution of gut alterations to MPP progression and their relationship with pulmonary pathology largely unexplored.

To address these gaps, we established a murine MPP model using a widely described repeated intranasal instillation protocol (three consecutive daily inoculations) [39]. Because a single challenge induces only mild inflammation due to the low virulence of MP in mice, repeated administration was employed to ensure robust colonization and consistent pathology. This model enables us to perform parallel multi-omics profiling across pulmonary and intestinal compartments. Specifically, we combined lung transcriptomics and metabolomics to characterize the local immune landscape and metabolic reprogramming, alongside fecal microbiome and metabolome sequencing to delineate gut microbial shifts and their functional metabolic outputs. By comprehensively interrogating both local and distal host responses, our study aims to: (i) reveal the pulmonary immune–metabolic interaction networks during MP infection; (ii) explore correlations between pulmonary and intestinal alterations to provide molecular clues for gut—lung axis involvement; and (iii) evaluate gut-derived microbial and metabolic features as candidate non-invasive biomarkers. This integrated approach offers a holistic, systems-level view of bridging immunology, metabolism, and microbial ecology, and provides a valuable experimental resource for identifying novel therapeutic targets and translational biomarkers for improved MPP management.

## 2. Materials and Methods

### 2.1. MP Culture and Concentration Determination

The MP standard strain Mut129 (CCTCC PB 2021038) was obtained from the China Center for Type Culture Collection (CCTCC) at Wuhan University, Wuhan, China. MP was cultured in complete medium consisting of PPLO powder (21 g/L, Hopebio, Qingdao, Shandong, China, REF#HB8475), yeast extract (4 g/L, OXOID Ltd., Basingstoke, UK, REF#LP0021B), 20% fetal bovine serum (FBS, Gibco Thermo Fisher Scientific, Waltham, MA, USA, REF#16000-044), 0.5% phenol red (4.8 mL/L, Solarbio Science & Technology Co., Ltd., Beijing, China, REF#G1230), 50% glucose (10 mL/L, Sinopharm Chemical Reagent Co., Ltd., Shanghai, China, REF#10010517), and penicillin G (1 g/L, Beyotime, Biotechnology, Shanghai, China REF#ST486-10g), with the pH adjusted to 7.9. The culture was incubated at 37 °C for 7 days until the phenol red indicator turned from red to orange, indicating acid production.

Given the small size of MP, conventional turbidimetric methods are unsuitable for quantification. Instead, the color change unit (CCU) method was employed to estimate MP concentration [40]. Briefly, 12 sterile EP tubes were labeled 1 through 12, each containing 900 μL of complete MP medium. A 100 μL aliquot of the bacterial suspension was serially diluted 10-fold across the tubes, with tube 12 serving as the blank control. Following incubation at 37 °C for approximately one week, the CCU titer was determined as the reciprocal of the highest dilution that exhibited a color change from red to orange/yellow, indicative of glucose fermentation and acid production. The MP concentration was calculated as 1 × 10^x^ CCU/mL, where ‘x’ represents the maximum dilution factor showing a positive color change [40]. The initial MP stock titer was determined to be 1 × 10^7^ CCU/mL by the CCU method (Figure S1). This stock was subsequently concentrated by centrifugation to prepare higher inoculum doses for the dose-ranging experiment.

### 2.2. Identification of MP by PCR

To confirm the identity of the MP strain used for inoculation, genomic DNA was extracted from the MP culture suspension using a commercial kit (TIANamp Bacteria DNA Kit, Tiangen Biotech Co., Ltd., Beijing, China). The PCR reaction mixture was prepared in a new PCR tube containing 10 μL of 2× Taq PCR Mix (Genesand, Wuhan, China), 1 μL each of forward primers (5′-ACCTCCTTTCTACGGAGTACAA-3′) and reverse primers (5′-GCATCCACTACAAACTCTT-3′), 6 μL of ddH2O, and 1 μL of the DNA of the lung. PCR amplification was performed under the following conditions: initial denaturation at 95 °C for 10 min, followed by 35 cycles of denaturation at 94 °C for 30 s, annealing at 58 °C for 25 s, and extension at 72 °C for 20 s, the final extension stage was carried out at a temperature of 72 °C for 10 min. 1 µL of the PCR product was used for nucleic acid gel electrophoresis.

### 2.3. Establishment of the Experimental MPP Model

Specific pathogen-free (SPF) male Balb/c mice, aged 4–5 weeks and weighing 14–16 g, were obtained from the Animal Experiment Center of Wuhan University (Wuhan, China). The mice were housed in individually ventilated cages (IVC) under controlled environmental conditions (temperature 20 ± 2 °C, humidity 50 ± 5%) within an Animal Biosafety Level 2 (ABSL-2) facility at the same institution. They were provided with sterilized SPF-grade chow and autoclaved water ad libitum. All experimental protocols were reviewed and approved by the Institutional Animal Care and Use Committee of the Animal Experiment Center of Wuhan University (approval number: WP20250189).

The mice were randomly divided into four groups, one control group (*n* = 3) and three experimental groups (*n* = 3 per group), which received 1 × 10^7^, 1 × 10^8^, or 1 × 10^9^ CCU/mL of MP suspension, respectively. To prepare higher inoculum concentrations, the 1 × 10^7^ CCU/mL MP stock was concentrated by centrifugation (4 °C, 15,000× *g*, 10 min) [41]. Specifically, the pellet from 20 mL of stock was resuspended in 2 mL or 200 μL of medium to obtain 1 × 10^8^ or 1 × 10^9^ CCU/mL, respectively. All suspensions were freshly prepared and verified by CCU titration before administration. After anesthesia induction with 3% isoflurane (RWD Life Science Co., Ltd., Shenzhen, Guangdong, China) at an air flow rate of 300 mL/min for 10 s, each mouse in the experimental groups received 50 μL of the corresponding MP suspension via intranasal instillation. The inoculum was fully inhaled through spontaneous breathing while the mouse was held upright for approximately 15 s. The control group received the same volume of sterile culture medium. Intranasal inoculation was repeated daily for three consecutive days (Day 2, Day 1, and Day 0). Body weight was monitored daily from Day 2 to Day 5. All mice were euthanized on Day 5 post-infection for sample collection. The mice were weighed daily, and a one-way analysis of variance was used to identify differences via the comparison of the three groups (10^7^, 10^8^, and 10^9^ CCU/mL) with the control group. Lung tissues were harvested and divided into three portions for histopathological examination, RNA extraction, and DNA extraction, respectively.

Based on the different pathological and inflammatory responses observed at various concentrations, we ultimately selected 1 × 10^8^ as the experimental concentration. Mice were randomly divided into two groups, a control group (*n* = 3) and an experimental group (*n* = 4) that received 1 × 10^8^ CCU/mL of MP suspension. The same protocol was followed. On day 5 post-infection, the right lung was lavaged via tracheal cannulation with two 0.5 mL aliquots of sterile PBS (total 1.0 mL), after ligation of the left bronchus. The recovered BALF (typically 0.6–0.8 mL) was centrifuged at 3000 rpm for 5 min at 4 °C, and the supernatant was stored at −80 °C for cytokine analysis. The left lung was excised for transcriptome sequencing (stored in RNA later). Fresh fecal samples were also collected from each mouse, immediately snap-frozen in liquid nitrogen, and stored at −80 °C for subsequent metabolomic analyses (LC-MS/MS, MAJORBIO, Shanghai, China).

The chosen sample sizes meet the minimum statistical requirements and were determined based on previous MPP murine studies with similar experimental designs [39]. Humane endpoints were predefined (≥20% weight loss, severe respiratory distress, or inability to access food/water), and mice were monitored twice daily. No animals reached these endpoints.

### 2.4. RT-qPCR Analysis of Cytokine Expression

To verify MP colonization in the lungs and quantify the expression of inflammation-related genes, PCR and RT-qPCR analyses were performed on lung tissue samples. For MP DNA detection, genomic DNA was extracted from lung tissue using a commercial kit (TIANamp Bacteria DNA Kit, TIANGEN), and PCR was performed using the same MP-specific primers described in Section 2.2. For cytokine expression analysis, total RNA was extracted from lung tissue with TRIzol™ reagent (Invitrogen, San Diego, CA, USA) per the manufacturer’s protocol. Purity and concentration were determined using a NanoDrop ND-1000 spectrophotometer(Thermo Fisher Scientific, Waltham, MA, USA), and RNA integrity was verified on an Agilent 2100 Bioanalyzer (Agilent Technologies, Santa Clara, CA, USA). cDNA was synthesized from 1 µg of RNA using the HiScript III Reverse Transcription Kit (Yeasen Biotechnology Co., Ltd., Shanghai, China) and diluted 1:3. Quantitative real-time PCR (RT-qPCR) was performed with Hieff™ SYBR Green Master Mix (Yeasen Biotechnology Co., Ltd., Shanghai, China) under the following conditions: 95 °C for 5 min, followed by 40 cycles of 95 °C for 10 s and 58 °C for 30 s. Each sample was analyzed in triplicate. The primer sequences used are listed in Table S1.

### 2.5. Histopathological Validation

The lung tissue specimens of mice were placed in 4% paraformaldehyde fixative solution (Biosharp, Beijing, China), then the fixed tissues were dehydrated, embedded in paraffin (Sinopharm Chemical Reagent Co., Ltd., Shanghai, China), and stained with hematoxylin-eosin (HE) (Servicebio, Wuhan, China). HE staining was performed on sections 5 μm thick. An upright microscope (Olympus BX53, Tokyo, Japan) was used to examine the pathological conditions of lung tissue in each group. For quantitative assessment, lung histopathological changes were evaluated using a semi-quantitative scoring system based on five parameters (listed in Supplementary Table S2), as previously described [42].

### 2.6. Detection of Pro-Inflammatory Cytokines in BALF

Cytokines in BALF were measured with a commercial kit (ABplex Mouse 15-Plex Custom Panel, ABclonal, Wuhan, China). Into each well, 5 μL of the bead mixture and 50 μL of standards or test samples were added, followed by incubation at 37 °C for 1 h and one magnetic wash. Subsequently, 50 μL of detection antibody solution was added, incubated at 37 °C for 30 min, and washed once. Then, 50 μL of fluorescein solution was added, and it was incubated at 37 °C for 15 min in the dark. After incubation, 55 μL of wash buffer was added to resuspend the beads, and the signals were acquired using the ABplex-100 system (ABclonal, Wuhan, China).

### 2.7. Flow Cytometric Analysis of BALF Immune Cells

Flow cytometry was used to detect macrophage cell changes in BALF. The BALF was centrifuged at 3000 rpm for 5 min, and the supernatant was discarded to obtain the cell pellet. Cells were stained with Live/Dead dye (BD Horizon™ Fixable Viability Stain 510: 1:100, cat#564406) (BD Horizon, Franklin Lakes, NJ, USA) and antibodies (ABflo^®^ 450 Rabbit an-ti-Mouse CD45 mAb: 1:200, ABclonal, cat#A27197; ABflo^®^ 488 anti-Mouse F4/80 mAb: 1:200, ABclonal, cat#A27479) (ABclonal, Wuhan, China). Staining was performed at 4 °C in the dark for 1 h, following the manufacturer’s recommended protocol (ABclonal, Wuhan, China), after which cells were washed twice with FACS buffer. The gating strategy was sequentially applied as: debris and aggregates were first excluded by FSC-A vs. SSC-A, followed by singlet discrimination using FSC-A vs. FSC-H; viable cell were selected as Live/Dead staining (negative); leukocyte were gated by CD45^+^; and alveolar macrophages were finally identified as CD45^+^F4/80^+^ cells (Figure S2). Data were analyzed using FlowJo (Version 10.8.1).

### 2.8. Transcriptomic Sequencing of Lung Tissue

Total RNA was isolated from lung tissues using TRIzol (Duesseldorf, Germany) and quantified/qualified with the NanoDrop2000 (Waltham, MA, USA) and Agilent 5300 Bioanalyser (Santa Clara, CA, USA). A total of 107.73 Gb of clean data was obtained, with each sample yielding at least 5.5 Gb of clean data and a Q30 base percentage of 96.52% or higher. Only high-quality RNA (OD260/280 1.8–2.2, OD260/230 ≥ 2.0, RIN ≥ 6.5, 28S:18S ≥ 1.0, >1 μg) was used for library construction. Stranded mRNA libraries were generated with the Illumina Stranded mRNA Prep Kit (illumina, San Diego, CA, USA) and sequenced on a NovaSeq 6000 (2 × 150 bp) (illumina, San Diego, CA, USA). Raw reads were processed with Fastp (https://github.com/OpenGene/fastp, access date on 1 February 2026), mapped to the reference genome (Mus_musculus, GRCm39) via HISAT2 (https://daehwankimlab.github.io/hisat2/, access date on 1 February 2026), and assembled with StringTie (Version 2.2.3). Gene expression was calculated as TPM using RSEM (http://deweylab.biostat.wisc.edu/rsem/, access date on 1 February 2026), and DEGs were screened by DESeq2 (FC > 1.20 or FC < 0.83, *p* < 0.05) (https://bioconductor.org/packages/release/bioc/html/DESeq2.html, access date on 1 February 2026). GO and KEGG enrichment were conducted with Goatools (https://github.com/tanghaibao/GOatools, access date on 1 February 2026) and KOBAS (Bonferroni-corrected *p* < 0.05) (http://kobas.cbi.pku.edu.cn/anno_iden.php, access date on 1 February 2026). Alternative splicing events were detected by Rmats (Version 4.1.2), covering exon inclusion/exclusion, alternative splice sites, and intron retention.

### 2.9. Metabolomic Profiling

LC-MS/MS analysis (MAJORBIO, Shanghai, China) was performed on mouse lung tissues and fecal samples. After instrument analysis, the raw data were processed using Progenesis QI (Waters Corporation, Milford, OH, USA) for peak detection, extraction, alignment, and integration. Compound identification was performed using the HMDB database (http://www.hmdb.ca/, access date on 20 February 2026), Metlin (https://metlin.scripps.edu/, access date on 20 February 2026), and Meiji’s inhouse library (MAJORBIO, Shanghai, China). To eliminate or reduce errors introduced during the experimental and analytical processes, the qualitatively analyzed data underwent preprocessing, which included removing features with missing values exceeding 20% within each group in the raw data, imputing missing values with the minimum value across all samples, and normalizing the response intensities of the mass spectrometry peaks to the total sum to obtain a normalized data matrix. Additionally, variables with a relative standard deviation (RSD) in QC samples exceeding 30% were removed, and the data were log10-transformed to obtain the final data matrix for subsequent analysis. Statistical analyses such as PCA and OPLS-DA were performed using the ropls package (Version 1.6.2) in R (Version 4.2.0). Metabolite annotation was conducted using the HMDB and KEGG databases (https://www.kegg.jp/kegg/pathway.html, access date on 1 February 2026), and pathway enrichment analysis was performed using the Python package (scipy.stats, Version 1.17.0). After data preprocessing, PLS-DA analysis was performed to visualize the separation between groups. Variables with VIP > 1, *p* < 0.05, |FC| ≥ 1.0 were considered DME. These DMEs were mapped to KEGG pathways. Pathway significance was determined based on the total number of metabolites mapped to that pathway and their respective significance; a pathway was considered disrupted if the number of significant metabolites was ≥2, the DA-score was >0.10, and *p* < 0.05.

### 2.10. 16S rRNA Sequence

After genomic DNA extraction using the E.Z.N.A.^®^ Bacterial DNA Kit (Omega Bio-tek, Norcross, GA, USA), the purified PCR products were subjected to library construction with the NEXTFLEX™ Rapid DNA-Seq Kit (illumina, San Diego, CA, USA). Raw paired-end reads were quality filtered using fastp (Version 0.23.4) to remove low quality bases (sliding window of 50 bp with average quality < 20) and reads containing > 5 ambiguous N bases. The clean PE reads were then merged using FLASH (Version 1.2.11) with a minimum overlap of 10 bp and a maximum mismatch ratio of 0.2. Samples were demultiplexed and sequence orientations were adjusted according to barcodes (0 mis-matches allowed) and primers (≤2 mismatches allowed). Operational taxonomic units (OTUs) were clustered from the merged sequences using USEARCH (Version 11) at 97% sequence identity, and chimeric sequences were removed. Taxonomic annotation was performed using the RDP Classifier (Version2.11) against the SILVA (Version 138.2) with a confidence threshold of 70%. For between group comparisons, *t* tests or rank sum tests were chosen based on data distribution and variance homogeneity. Overall differences in bacterial community structure between groups were assessed using ANOSIM/Adonis, and community compositions were visualised by bar plots and heatmaps. Differential species were identified using Wilcoxon test, and key discriminant taxa were further screened by LEfSe analysis (LDA score > 2, *p* < 0.05).

### 2.11. Statistics

Statistical analyses were performed using GraphPad Prism version 10.0 (GraphPad Software, Inc., San Diego, CA, USA). Data are presented as mean ± SD. Group comparisons were performed using independent-samples *t*-test (two groups) or one-way ANOVA with Tukey’s post-hoc test (≥three groups). Correlation analyses were performed using Pearson’s correlation coefficient, without adjustment for multiple comparisons. All statistical tests were two-sided, and *p* < 0.05 was considered statistically significant, with * *p* < 0.05, ** *p* < 0.01, *** *p* < 0.001. All statistical methods have been clearly described in the revised.

To minimize potential bias, investigators performing histopathological scoring, flow-cytometry gating and analysis, and bioinformatic analyses were blinded to group allocation. Group codes were revealed only after all data were acquired and analyzed.

## 3. Results

### 3.1. Successful Establishment of the MPP Model

**Figure 1:**
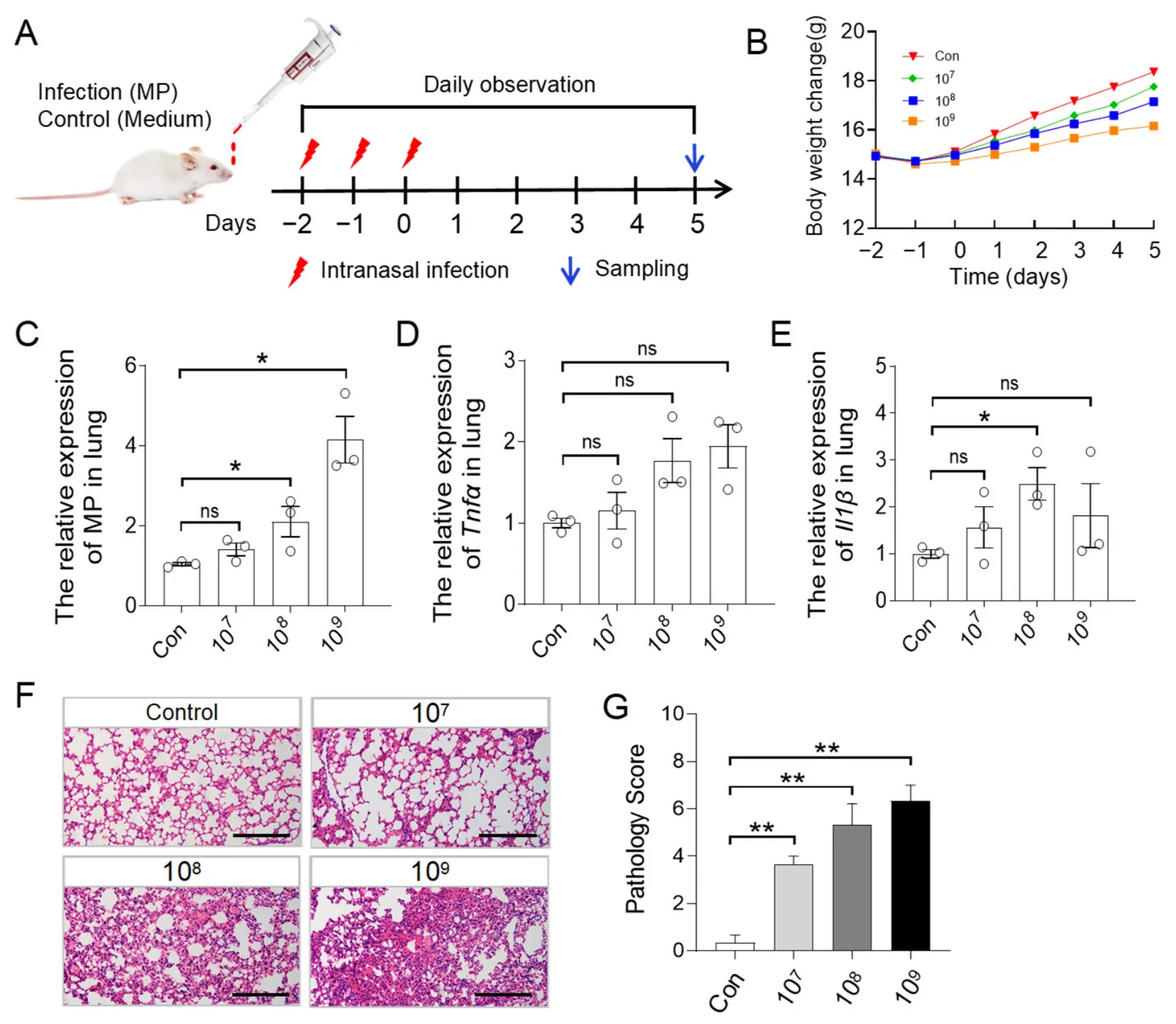
**Establishment of the MPP mouse model.** (**A**) Animal experimental design. Mice received intranasal instillations on Days 2, 1, and 0 and were euthanized on Day 5. (**B**) Body weight changes of the mice from Day 2 to Day 5 post-infection. (**C**) Relative MP load in lung tissues measured by PCR. (**D**) Relative *Tnfα* mRNA expression in lung tissues. (**E**) Relative *Il1β* mRNA expression in lung tissues. (**F**) HE staining of the lung tissue samples collected from each group. Scale bar: 100 μm. (**G**) Histopathological scores of lung tissues from each group. Data are presented as mean ± SD (*n* = 3). * *p* < 0.05, ** *p* < 0.01; ns, not significant.

The CCU titer of MP was determined to be 10^7^ CCU/mL by observing the highest dilution that exhibited a color change (Figure S1). The experimental implementation is illustrated as shown in Figure 1A. After the intranasal challenge, a significant decrease in the weight of mice in the infected group was observed (Figure 1B), with exhibited poor mental status, lethargy, and listlessness, dull and erect hair with obvious piloerection, reduced locomotion, secretions around the mouth and nose, whereas the control group showed no abnormal clinical symptoms. Compared with the blank control group (Con), the expression levels of MP in all model groups increased; among them, the differences in the 10^8^ and 10^9^ CCU/mL groups were statistically significant (Figure 1C). Similarly, *Tnfα* expression increased in the model groups, although the differences were not statistically significant compared with the Con group (Figure 1D). *Il1β* expression levels were elevated in all model groups relative to the Con group, with a statistically significant difference observed in the 10^8^ CCU/mL group (Figure 1E). Histological examination (H&E staining) of lung tissues revealed that the 10^7^ CCU/mL group exhibited mild inflammatory cell infiltration and slight thickening of the alveolar septa in lung tissues. The 10^8^ and 10^9^ CCU/mL groups showed extensive inflammatory cell infiltration, along with widespread alveolar collapse, thickening of the alveolar septa, and neutrophilic infiltration. In addition, peribronchial inflammatory cell infiltration, interstitial edema, and hemorrhage were noted (Figure 1F). In summary, intranasal administration of MP successfully recapitulated the key features of MPP in Balb/c mice, including detectable MP colonization, interstitial pneumonia-like histopathology, and enhanced pulmonary inflammatory responses. Following dose comparison, the 1 × 10^8^ CCU/mL concentration was determined to be the most suitable for subsequent mechanistic investigations.

### 3.2. BALF Pro-Inflammatory Cytokines and Immune Cells Verification in MPP Mouse Model

**Figure 2:**
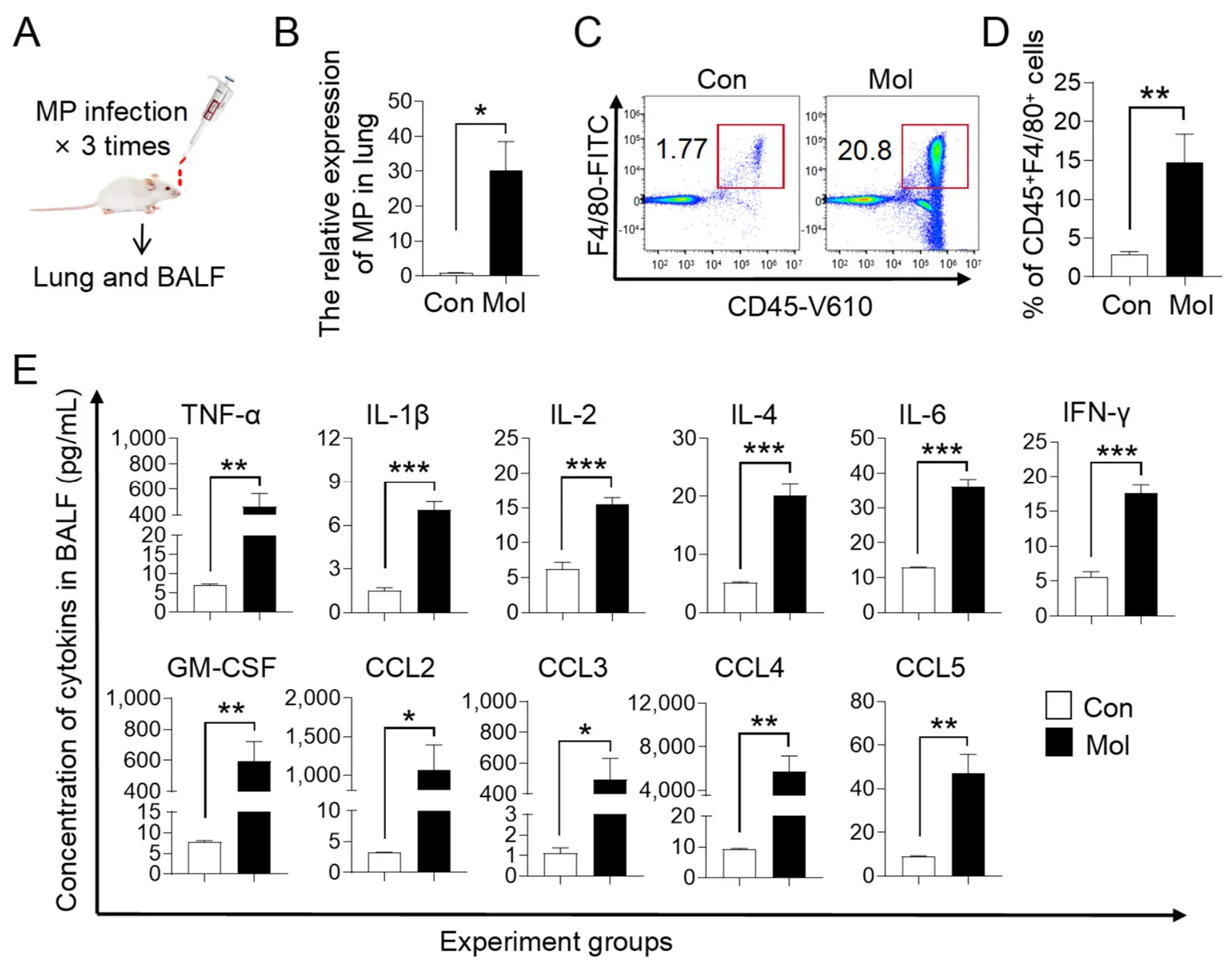
**MP induces inflammation in the lung.** (**A**) Schematic of BALF collection from mice. (**B**) MP colonization in lung tissues. qPCR analysis of MP DNA levels in lung tissues. (**C**) Representative flow cytometry scatter plots showing CD45^+^F4/80^+^ alveolar macrophages in BALF of control (Con) and MP-infected (Mol) groups. Red boxes indicate the percentage of F4/80^+^ CD45^+^ macrophages within the BALF cell pellet. (**D**) Quantification of alveolar macrophage percentages among the BALF cell pellet. (**E**) Cytokines and chemokine concentrations in BALF of mice. Data are presented as or mean ± SD (Con = 3, Mol = 4). * *p* < 0.05, ** *p* < 0.01, *** *p* < 0.001.

qPCR analysis of lung tissues from the final experimental groups confirmed successful MP colonization, with MP DNA levels significantly elevated in the Mol group compared to the Con group (Figure 2A,B). Based on this MP infection model, flow cytometry was performed to assess alveolar macrophage infiltration in BALF (Figure 2C,D and Figure S2). The proportion of alveolar macrophages (gated as CD45^+^F4/80^+^ cells) increased significantly in the MPP group compared to the control group (14.79 ± 8.76% vs. 2.86 ± 0.98%, *p* < 0.01) (Figure 2C,D), indicating that MP infection promotes substantial macrophage recruitment in the lung microenvironment. The concentrations of cytokines and chemokines in BALF were determined using multiparametric flow cytometry analysis. As presented in Figure 2E, the MPP group exhibited significantly elevated levels of multiple pro-inflammatory cytokines, including TNF-α, IL-1β, IL-2, IL-4, IL-6, IFN-γ, and GM-CSF, as well as chemokines such as CCL2, CCL3, CCL4, and CCL5, compared to the control group, demonstrating that MP infection triggered a robust and broad-spectrum inflammatory response in the lung.

### 3.3. Transcriptomic Profiling Reveals Activation of Anti-Infective Immune and Inflammatory Responses in MP-Infected Lung

**Figure 3:**
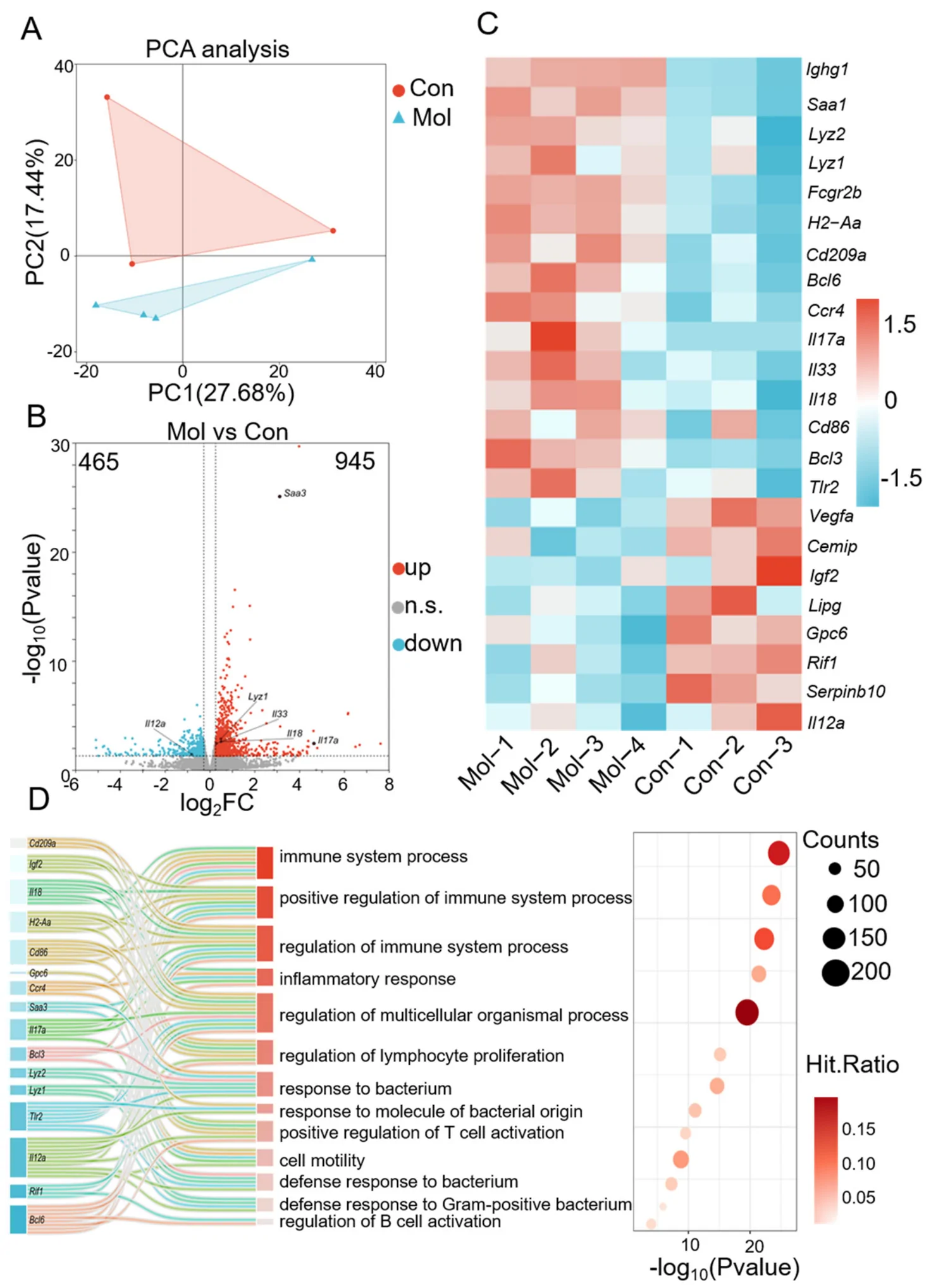
**Transcriptomic profiling reveals activation of anti-infective immune and inflammatory responses in MP-infected lung.** (**A**) PCA analysis of DEGs between Con and Mol groups. Different colors represent different treatment groups: red is Con group, and blue is Mol group; the lines connect the individual samples. (**B**) Volcano plots of DEGs between Con and Mol groups. Red indicates genes with increased expression (up), blue indicates genes with decreased expression (down), and gray indicates genes with no significant change (n.s.). The lines indicate the corresponding gene names. (**C**) Heatmap of DEGs between Con and Mol groups. (**D**) Sankey bubble chart of Gene Ontology (GO) enrichment analysis for DEGs. The blue borders represent genes, the red borders represent GO pathways, and the differently colored lines represent the GO pathways in which different genes are involved.

To elucidate the pulmonary transcriptional response triggered by MP infection, we performed transcriptomic profiling. Principal component analysis (PCA) revealed a separation of samples from the control (Con) and MP-infected (Mol) groups along the principal components, indicating marked divergence in transcriptomic profiles (Figure 3A), with 945 upregulated and 465 downregulated genes identified (Figure 3B). The heatmap showed elevated expression of antimicrobial genes (*Saa3*, *Lyz1*, *Lyz2*) in the Mol group, suggesting that MP invasion elicited an antimicrobial immune response. Concurrently, pro-inflammatory cytokine genes (*Il17a*, *Il18*, *Il33*) were significantly upregulated, whereas the anti-inflammatory gene *Il12a* was downregulated, indicative of inflammatory activation. T/B lymphocyte-related genes (*Bcl3*, *Tlr2*, *Cd86)* were also coordinately upregulated, foreshadowing the initiation of adaptive immunity. Conversely, several genes involved in cell growth and apoptosis regulation (*Serpinb10*, *Vegfa*, *Igf2*) were markedly downregulated, suggesting that infection disrupted cell growth homeostasis and may lead to lung structural damage (Figure 3C). These differentially expressed genes (DEGs) were significantly enriched in biological processes such as “immune system process”, “inflammatory response”, “positive regulation T cell activation”, and “defense response to bacterium” (Figure 3D). KEGG pathway enrichment analysis revealed that DEGs were significantly enriched in pathogen recognition and immunity pathways, including “cytokine–cytokine receptor interaction”, “lysosome”, “Th17 cell differentiation”, “B cell receptor signaling”, “NF-κB signaling”, as well as “Fc gamma R-mediated phagocytosis” and “antigen processing and presentation”. Notably, the DEGs were also enriched in disease-related pathways such as tuberculosis, Staphylococcus aureus infection, and coronavirus disease (COVID-19), implying that *M. pneumoniae* infection shares certain molecular hallmarks with these diseases (Figure S3). Collectively, these findings demonstrate that MP infection activates anti-infective immune and inflammatory signaling networks in the lung.

### 3.4. Metabolic Profiling Reveals Disrupted Lung Homeostasis Induced by MP Infection

**Figure 4:**
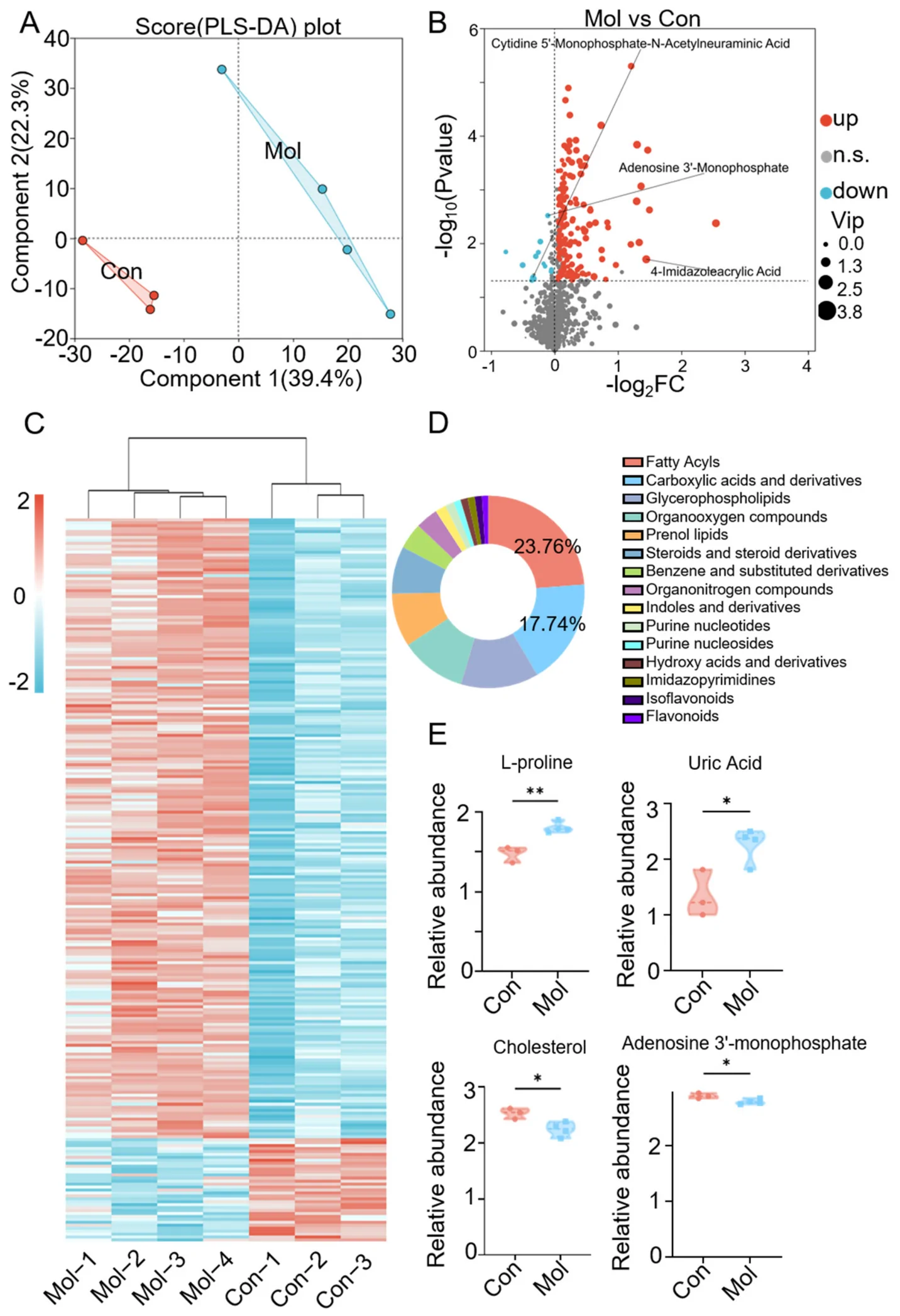
**Metabolic profiling reveals disrupted lung homeostasis induced by MP infection.** (**A**) Partial least squares discriminant analysis between Con and Mol groups. Different colors represent different treatment groups: red is Con group, and blue is Mol group; the lines connect the individual samples. (**B**) The volcano plot shows the types of metabolites that are significantly increased (red) or decreased (blue) between Con and Mol groups, and gray indicates metabolites with no significant change (n.s.). These lines label the corresponding metabolite names. (**C**) Clustering heatmap of lung metabolites in Con and Mol groups. (**D**) Classify the differential metabolites according to the Human Metabolome Database. (**E**) The distribution of differential metabolites with significant anti-inflammatory or pro-inflammatory effects between the two groups. * *p* < 0.05, ** *p* < 0.01.

To investigate the impact of MP infection on metabolic homeostasis in lung tissue, an untargeted metabolomics approach was employed. Partial least squares discriminant analysis (PLS-DA) coupled with permutation testing revealed a separation of metabolic profiles between the control (Con) and MP-infected (Mol) groups (Figure 4A). These results demonstrate that MP infection substantially alters the metabolite composition of the lung, with a predominance of upregulated metabolites (Figure 4B). This metabolic phenotypic divergence was further corroborated by a heatmap of differential metabolites (Figure 4C). Chemical class annotation indicated that fatty acids constituted the most abundant class of altered metabolites, while carboxylic acid derivatives were also significantly perturbed (Figure 4D). Meanwhile, pro-inflammatory metabolites, including L-proline and uric acid, were significantly increased in the Mol group, whereas anti-inflammatory metabolites, adenosine 3′-monophosphate (3′-AMP) and cholesterol, were markedly reduced after infection. These findings indicated that mycoplasma infection drives a shift toward a pro-inflammatory metabolic signature in the lung, and such metabolic disturbances may contribute to the progression of pulmonary inflammatory responses. Collectively, these findings suggest that MP invasion profoundly disrupts the metabolic homeostasis of the lung.

### 3.5. Alterations in Metabolites of MP-Infected Lung Were Correlated with Changes in the Expression of Inflammation-Related Genes

**Figure 5:**
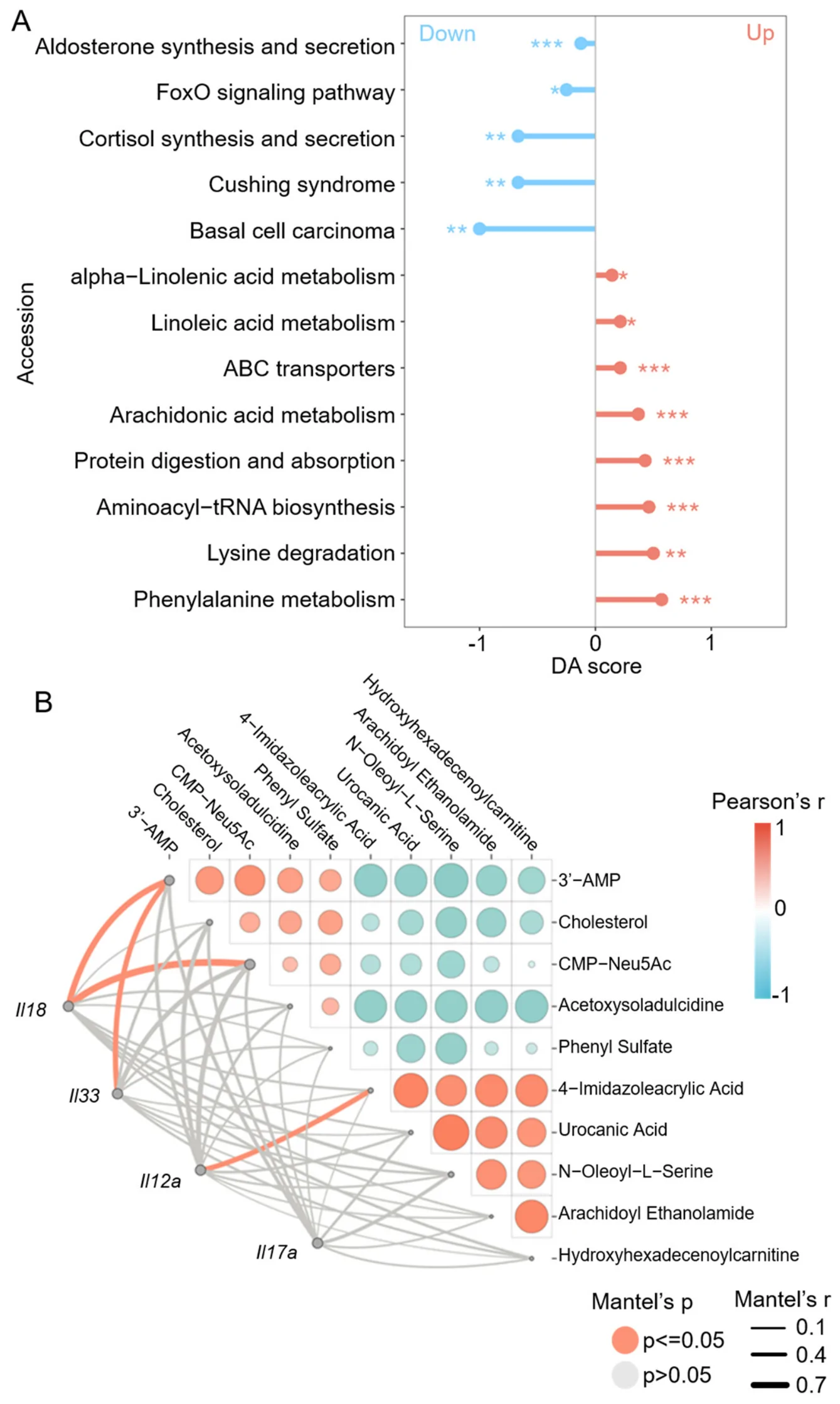
**Alterations in metabolites of MP-infected lung were correlated with changes in the expression of inflammation-related genes.** (**A**) Differential activity score (DA score) plot for pathway analysis of differential metabolites. Significantly enriched metabolic pathways with upregulated and downregulated signals after infection are presented. * *p* < 0.05, ** *p* < 0.01, *** *p* < 0.001. (**B**) Correlation network diagram of pulmonary differential metabolites and transcript-level inflammatory cytokines (*Il18*, *Il33*, *Il12a*, *Il17a*). The Pearson correlation coefficient is used for correlation analysis, and the Euclidean distance is used for all distance calculations. *p* values are nominal and were not corrected for multiple comparisons.

To elucidate the crosstalk between metabolic reprogramming and immune transcriptional regulation, we first performed KEGG pathway enrichment analysis of differentially accumulated metabolites. The results showed that metabolites in pathways such as arachidonic acid metabolism, α-linolenic acid metabolism, linoleic acid metabolism, ABC transporters, phenylalanine metabolism, and lysine degradation were predominantly upregulated, whereas those in endocrine-related pathways, including aldosterone synthesis and secretion and cortisol synthesis, were generally downregulated (Figure 5A). Given the close involvement of arachidonic acid, α-linolenic acid, and linoleic acid metabolism in inflammatory responses, we subsequently conducted a Mantel test to assess the associations between the top five most upregulated and the top five most downregulated metabolites and the transcript-level associations inflammatory cytokines *Il12a*, *Il17a*, *Il18*, and *Il33*. The analysis revealed that the pro-inflammatory gene *Il18* was correlated with the downregulated metabolites adenosine 3′-monophosphate (3′-AMP) and cytidine 5′-monophosphate-N-acetylneuraminic acid (CMP-Neu5Ac), and *Il33* also exhibited a positive correlation with 3′-AMP. In contrast, the anti-inflammatory gene *Il12a* was correlated with the upregulated metabolite 4-imidazoleacrylic acid (Figure 5B). These findings promote the secretion of pro-inflammatory cytokines, whereas the elevation of 4-imidazoleacrylic acid suppresses anti-inflammatory cytokine expression, thereby exacerbating pulmonary inflammation. Collectively, MP-induced metabolic dysregulation appears to coordinately modulate immune gene expression, jointly contributing to the inflammatory pathology of lung infection.

### 3.6. Dysbiosis of Fecal Microbiota and Metabolic Disruption Correlate with Inflammation upon M. pneumoniae Infection

**Figure 6:**
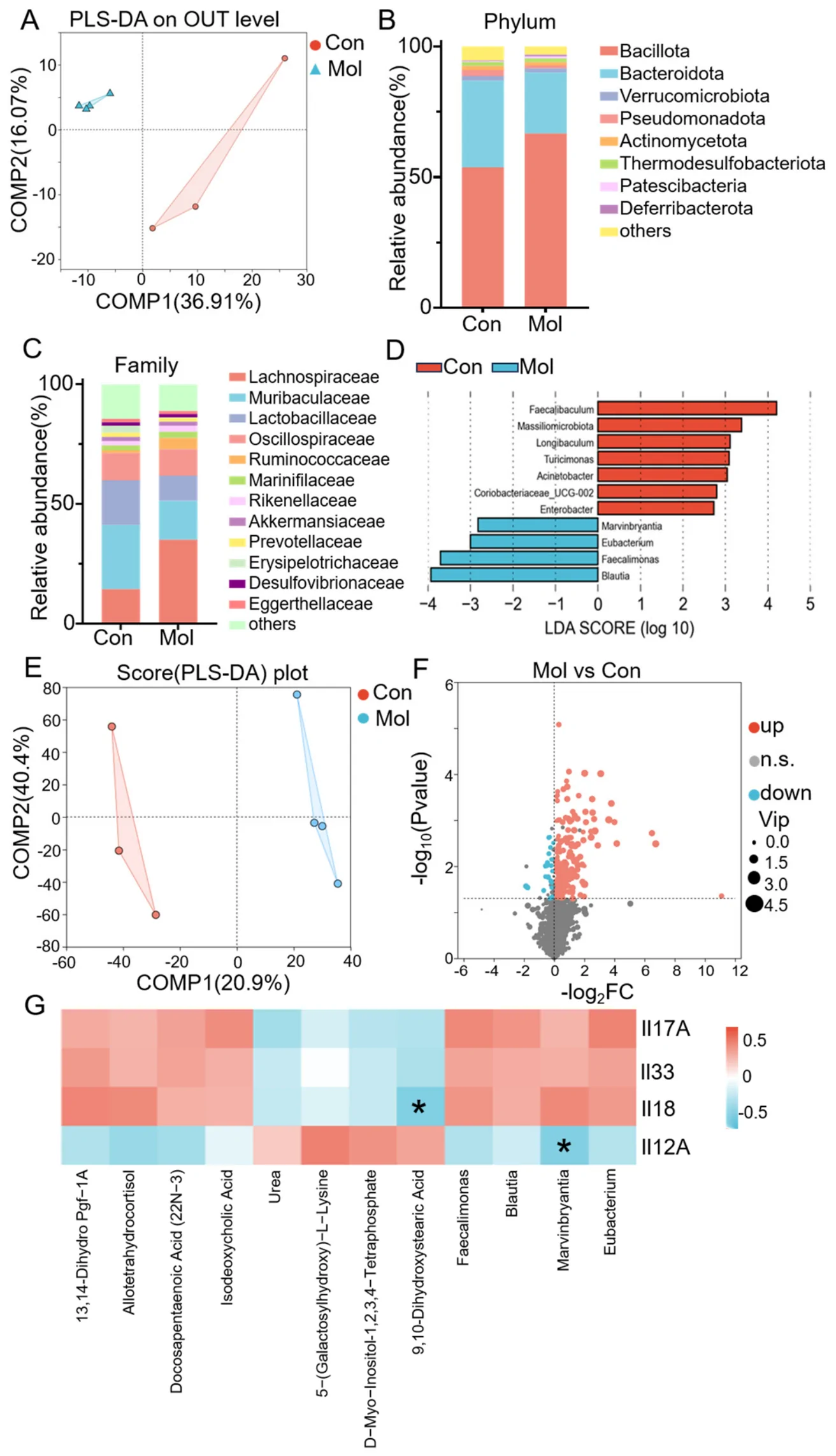
**Dysbiosis of the colonic microbiota and fecal metabolic disruption correlate with inflammation upon MP infection.** (**A**) PLS-DA score plot at the OTU level of the colon. Different colors represent different treatment groups: red is Con group, and blue is Mol group; the lines connect the individual samples. (**B**) Stacked bar chart showing the relative abundance of gut microbiota at the phylum level. (**C**) Stacked bar chart showing the relative abundance of gut microbiota at the family level. (**D**) Linear discriminant analysis (LDA) score plot of genera with significant differences between Con and Mol groups revealed by LEfSe analysis. (**E**) PLS-DA score plot based on untargeted metabolomics of feces. Different colors represent different treatment groups: red is Con group, and blue is Mol group; the lines connect the individual samples. (**F**) Volcano plot of fecal metabolites between Con and Mol groups. Red indicates metabolites with increased expression (up), blue indicates metabolites with decreased expression (down), and gray indicates metabolites with no significant change (n.s.). (**G**) Correlation heatmap of significantly differentially fecal metabolites, microbial gene levels, and transcript-level inflammatory cytokines (*Il17a*, *Il33*, *Il18*, *Il12a*). The Pearson correlation coefficient is used for correlation analysis, and the Euclidean distance is used for all distance calculations. *p* values are nominal and were not corrected for multiple comparisons. * *p* < 0.05.

In addition to profiling lung metabolites, we examined the impact of *Mycoplasma pneumoniae* infection on the fecal microbiome and fecal metabolome. The infection altered the gut microbial community structure, as evidenced by a clear separation in PLS-DA (Figure 6A). At the phylum level, the infected group exhibited an increased relative abundance of Bacillota and a decreased abundance of Bacteroidota (Figure 6B). At the family level, Lachnospiraceae and Ruminococcaceae were enriched, whereas Muribaculaceae and Lactobacillaceae were depleted (Figure 6C). Linear discriminant analysis (LDA) further identified significant enrichment of *Marvinbryantia*, *Eubacterium*, *Faecalimonas*, and *Blautia* in the Mol group (Figure 6D). These findings indicate that MP infection disrupts intestinal microbial homeostasis. Fecal metabolomic profiling revealed marked post-infection changes (Figure 6E and Figure S4A), with metabolites significantly upregulated or downregulated, and the majority of these differential metabolites belonged to the class of carboxylic acids and derivatives (Figure 6F and Figure S4B). To explore potential gut–lung axis interactions, we performed an association analysis linking the top four endogenous differential metabolites and the differentially abundant genera with the lung transcript-level associations of inflammatory cytokines. The results showed that the pro-inflammatory cytokine *Il18* was correlated with the downregulated metabolite 9,10-dihydroxystearic acid, whereas the anti-inflammatory cytokine *Il12a* was correlated with the abundance of *Marvinbryantia* (Figure 6G). Collectively, these data demonstrate that MP infection not only affects the lung but also disturbs fecal microbial homeostasis and alters the fecal metabolite profile. The downregulation of 9,10-dihydroxystearic acid and the enrichment of *Marvinbryantia* in feces may both be linked to the inflammatory response.

## 4. Discussion

In the present study, we successfully established a murine model of MPP via intranasal instillation of MP for three consecutive days. The model was confirmed by multiple lines of evidence, including positive MP detection in lung tissues, typical interstitial pneumonia histopathology, and elevated expression of pro-inflammatory cytokines. Among the three tested inoculum doses, the 10^7^ CCU/mL group exhibited only mild inflammatory cell infiltration and modest cytokine upregulation, failing to recapitulate the typical pathological features of MPP. In contrast, the 10^8^ CCU/mL group demonstrated extensive inflammatory cell infiltration, alveolar septal thickening, peribronchial inflammation, and significantly elevated *Il1β* expression—collectively mirroring the characteristic interstitial pneumonia observed in clinical MPP patients [26,43]. Although the 10^9^ CCU/mL group also displayed pronounced pathological changes, the 10^8^ dose was an optimal challenge dose that ensures both sufficient disease manifestation and animal welfare compliance, a consideration often overlooked in previous MPP mouse studies [44]. Therefore, the 1 × 10^8^ CCU/mL concentration was determined to be the most suitable for subsequent mechanistic investigations.

Our model reproduced the pulmonary cytokine/chemokine signature of pediatric MPP, with broad elevation of inflammatory mediators in BALF (most notably IL-1β), consistent with clinical findings [43]. Flow cytometric analysis revealed a significant increase in alveolar macrophage proportions in the BALF of infected mice. Previous studies have shown that alveolar macrophages serve as the first line of defense and play a dual role in MP infection-mediating bacterial clearance via phagocytosis while also contributing to excessive inflammation through pro-inflammatory cytokine release [14,16]. Our study observed macrophage expansion, likely reflecting both monocyte recruitment and local proliferation [44]. Collectively, these findings confirm that the MP infection model effectively establishes a pulmonary inflammatory response that mirrors key aspects of the clinical setting. To further explore the underlying regulatory mechanisms, we conducted integrated transcriptomic and metabolomic analyses to systematically profile the global immune and metabolic disturbances during MP infection, with a particular focus on their potential crosstalk through the gut–lung axis. Multiple studies have revealed that the levels of IL-1β, IL-6, IL-8, IL-10, IFN-γ, and TNF-α in BALF are positively correlated with mycoplasma load following infection [45,46,47]. In the present study, transcriptomic analysis further revealed that the expression of pro-inflammatory cytokines *Il17a*, *Il18*, and *Il33* was significantly upregulated in infected lungs, whereas the anti-inflammatory cytokine *Il12a* was markedly downregulated. Relevant studies indicate that *Il17a*, and *Il18*, are central to neutrophil recruitment and Th1 polarization and have been linked to disease severity and MP-associated complications [48,49], *Il33* serves as an epithelial-derived alarmin that amplifies type 2 inflammation [50]. Furthermore, elevated *Il33* has been identified as a prognostic factor in idiopathic pulmonary fibrosis [51], and upregulation of *Il17a* has been linked to the progression of MP-associated bronchiolitis obliterans [48], underscoring the potential relevance of these cytokines to fibrotic sequelae. As previously reported, *Il12* levels were decreased in pediatric patients with MP and EBV co-infection and were associated with poorer prognosis [52]. These findings are consistent with previous transcriptomic studies in pediatric MPP BALF [25,32,53], and were significantly enriched in the NF-κB signaling pathway. As a core transcription factor that mediates cellular responses to internal and external stimuli, NF-κB plays a pivotal role in immunity, cell survival, proliferation, and stress responses. It also acts as a critical regulator driving the expression of pro-inflammatory cytokines (TNF-α, IL-6, and IL-8), chemokines, and adhesion molecules, thus forming an essential line of host anti-infective defense and tissue repair [54,55]. Collectively, these findings suggest that MP infection may drive lung injury through activation of immune cascades coupled with suppression of tissue repair programs, offering a novel molecular perspective on the chronicity and sequelae of MPP.

However, pulmonary inflammation triggered by infection does not arise solely from the activation of immune transcriptional pathways; metabolic dysregulation may also participate in modulating inflammatory signals and thereby mediate the pathological progression of lung. Previous studies have shown that levels of pro-inflammatory metabolites in BALF are significantly elevated, whereas those of anti-inflammatory metabolites decline after mycoplasma infection [56]. In line with this, our study detected increased levels of pro-inflammatory metabolites, including L-proline and uric acid, alongside reduced levels of metabolites with anti-inflammatory properties, such as adenosine 3′-AMP and cholesterol. In addition, lipid metabolic pathways involving arachidonic acid, α-linolenic acid, and linoleic acid were synchronously activated, and the resultant eicosanoid lipid mediators are well-recognized drivers of inflammatory responses [57]. The downregulation of metabolites in the aldosterone and cortisol synthesis pathways is consistent with reports of adrenal dysfunction in critical illness [58,59], and a serum metabolomic study in MP-infected mice further identified cortisol as a significantly altered metabolite, with steroid hormone synthesis among the perturbed pathways [27]. Furthermore, correlation analysis between metabolites and inflammatory cytokines showed that the downregulation of endogenous metabolites 3′-AMP and CMP-Neu5Ac was significantly associated with the upregulation of pro-inflammatory cytokines *Il18* and *IL-33Il33*. This synergistic perturbation at both the metabolic and transcriptional levels provides a mechanistic explanation from a metabolic perspective for the sustained progression of inflammation in the MP group. Meanwhile, it indicates that key pulmonary metabolites closely linked to inflammatory pathways may serve as tissue biomarkers reflecting pulmonary inflammatory activity, thus offering additional support for the assessment of disease severity.

Gut microbiota, a microecosystem colonizing the surface of the host intestinal mucosa, serves as a key regulator of host physiological homeostasis, with its primary functions manifested in the fundamental regulation of local intestinal barrier integrity, immune development, and metabolic homeostasis [60,61]. However, when disrupted by external stress, gut microbial homeostasis is compromised. In the present study, we also observed alterations in gut microbial composition following MP infection. These findings align with prior MPP studies reporting depletion of protective commensals (Lactobacillaceae) and enrichment of opportunistic taxa [33,39,62]. Our phylum-level Bacillota/Bacteroidota shift aligns with Wang et al. [24], who reported an elevated Firmicutes/Bacteroidetes ratio in MPP, but diverges from Xu et al. [34], who observed the opposite trend. This discrepancy is likely attributable to differences in sample type (children versus mice) and disease severity, as acute infection may drive more pronounced phylum-level alterations than mixed or convalescent stages. At the genus level, this was evidenced by significantly increased relative abundances of *Faecalimonas*, *Blautia*, *Marvinbryantia*, and *Eubacterium* in feces after infection. *Blautia* and *Eubacterium* have a pro-inflammatory role, while *Marvinbryantia* and *Faecalimonas* link to metabolic disturbances and inflammation [34,62]. Similar phenomena have been reported in prior studies: the abundance of *Marvinbryantia* is elevated in the gut microbiota of patients with colorectal cancer, which may be associated with tumorigenesis [63]. Furthermore, we found that the relative abundance of this genus was negatively correlated with the expression level of the anti-inflammatory cytokine *Il12a*. In other words, the increased abundance of intestinal *Marvinbryantia* after MP infection negatively regulates *Il12a*, dampens the host’s anti-inflammatory response, and subsequently exacerbates inflammatory injury. We acknowledge that the correlations between metabolites/microbiota and the four cytokines (*Il18*, *Il33 Il12a*, *Il17a*) presented in Figure 5B and Figure 6G were based on transcriptomic data (mRNA levels) rather than validated protein measurements. While RNA-seq provides a genome-wide survey of transcriptional changes, it does not necessarily reflect protein abundance or biological activity. However, future studies should validate these transcript-level associations by targeted protein assays (e.g., ELISA or expanded multiplex panels) to confirm whether the observed correlations are maintained at the protein level.

Our finding that fecal 9,10-Dihydroxystearic Acid (DHSA) was downregulated in MP-infected mice, along with its negative correlation with IL-18, suggests a potential link to PPAR-γ-mediated macrophage regulation. Given that DHSA activates PPARs and PPAR-γ skews macrophages toward an M2 anti-inflammatory phenotype while suppressing M1 pro-inflammatory signals [64]. The alterations in gut microbiota and fecal metabolic profiles induced by MP infection are not merely secondary incidental phenomena of infection but represent a critical component involved in the progression of pulmonary inflammation under the regulatory network of the gut–lung axis. From a clinical perspective, fecal samples have the advantages of easy accessibility and suitability for continuous monitoring. The differentially expressed bacterial genera and metabolites in fecal samples, along with inflammatory factors in lung tissue, provide evidence supporting the gut–lung axis [33] and suggest that these may serve as potential markers for assessing the severity of pulmonary inflammation. At the same time, these findings offer preliminary insights for future exploration of adjunctive treatments for MPP from the perspective of the gut microbiome (probiotic or metabolite interventions). However, this approach still requires further in vivo experimental validation.

While our study provides initial insights, it is important to note that only male animals were examined. Given that sex-related variations in immune responses are well documented, the extent to which our observations apply to females remains to be determined. Ideally, subsequent studies that include both sexes will further address this question. It must be acknowledged that the sample size in this animal study was relatively small, which may have reduced statistical power and limited the generalizability of the findings. Independent validation in larger animal cohorts and clinical populations is needed in the future to further investigate the candidate biomarkers and the immune–metabolic regulatory network identified in this study. Moreover, the correlative findings between metabolites/microbiota and cytokines (Figure 5B and Figure 6G) were not adjusted for multiple comparisons; therefore, they should be interpreted as exploratory and require confirmation in future studies. Furthermore, it is important to acknowledge that the present multi-omics approach is primarily correlative and hypothesis-generating, rather than mechanistically confirmatory. While we identified several candidate metabolites (e.g., 3′ AMP, 4 imidazoleacrylic acid) and microbial taxa (e.g., *Marvinbryantia*) that are significantly associated with pulmonary transcript-level inflammatory cytokines, the causal relationships between these candidates and disease progression cannot be established from our current data. Functional interventions—such as metabolite supplementation, gene knockout, or fecal microbiota transplantation—are required in future studies to rigorously test the causative roles of these candidates in the gut–lung axis. Nevertheless, we believe that the systematic landscape provided here offers a valuable resource for prioritizing targets and designing subsequent mechanistic experiments.

## 5. Conclusions

In summary, MP infection drives pulmonary inflammation and macrophage overactivation, alongside transcriptomic imbalance and metabolic remodeling in the lung. Exploratory correlations between key metabolites (3′-AMP, 4-imidazoleacrylic acid) and inflammatory cytokines (*Il12a* and *Il18*) suggest metabolic reprogramming may modulate immunity. Concentric gut dysbiosis (*Marvinbryantia* enrichment) and altered fecal metabolites (decreased 9,10-dihydroxystearic acid), also linked to *Il12a* and *Il18*, provide preliminary support for gut–lung axis involvement. Our findings delineate immune–metabolic perturbations in MPP and provide exploratory evidence that fecal microbial and metabolite signatures may serve as candidate auxiliary diagnostic markers and potential therapeutic targets.

## Data Availability

The raw sequencing datasets generated and analyzed during the current study are available in the NCBI Sequence Read Archive (SRA) repository. The raw data of RNA-seq of lung were deposited in the NCBI SRA database under accession number PRJNA1499669 (https://www.ncbi.nlm.nih.gov/bioproject/PRJNA1499669, accessed on 23 July 2026). The raw data of the microbiome of faces were deposited in the NCBI SRA database under accession number PRJNA1501672 (https://www.ncbi.nlm.nih.gov/bioproject/PRJNA1501672, accessed on 29 July 2026). The raw data of the metabolome of the lung were deposited in the CNCB OMIX database under accession number OMIX018934. The raw data of the metabolome of feces were deposited in the CNCB OMIX database under accession number OMIX018935.

## Supplementary Materials

The following supporting information can be downloaded at: https://www.mdpi.com/article/10.3390/biology15171469/s1, Figure S1: Determination of MP concentration and identification. (A) CCU titer determination of *M. pneumoniae* by color change method, suggesting MP concentration was determined to be 10^7^ CCU/mL. (B) PCR identification of MP in lung tissues. C ELISA identification of MP antigen in lung tissue lysates. Data are presented as mean ± SD. Figure S2: Gating strategy for alveolar macrophage identification in BALF. Cells were first gated on forward scatter area (FSC-A) versus side scatter area (SSC-A) to exclude debris, followed by doublet discrimination using FSC-A versus FSC-H to select singlets. Live cells were then gated as negative for Live-Dead Aqua staining. Among live cells, leukocytes were identified as CD45^+^ cells, and alveolar macrophages were further defined as CD45^+^F4/80^+^ cells. Fluorescence minus one (FMO) were used to define the positive gates for CD45 and F4/80. Representative plots are shown with the percentages of cells in each gate. Figure S3: Transcriptomic Profiling Reveals Activation of Anti-Infective Immune and Inflammatory Pathways in MP-Infected Lung. KEGG pathway enrichment analysis of differentially expressed genes in lung tissues upon *Mycoplasma pneumoniae* infection. Figure S4: Dysbiosis of Fecal Microbiota and Metabolic Disruption Correlate with Inflammation upon MP Infection. (A) Hierarchical clustering heatmap of fecal differential metabolites in Con and Mol group. (B) Classify the differential metabolites of feces according to Human Metabolome Database. Table S1: Primer sequences used for mRNA RT-qPCR. Table S2: Histopathological scoring system for lung tissues. Table S3: The number and percent of metabolites in class.

## References

[1] Foy H.M. (1993). Infections caused by *Mycoplasma pneumoniae* and possible carrier state in different populations of patients. Clin. Infect. Dis..

[2] Brown R.J., Nguipdop-Djomo P., Zhao H., Stanford E., Spiller O.B., Chalker V.J. (2016). *Mycoplasma pneumoniae* epidemiology in England and Wales: A national perspective. Front. Microbiol..

[3] Xu Y., Yang C., Sun P., Zeng F., Wang Q., Wu J., Fang C., Zhang C., Wang J., Gu Y. (2024). Epidemic features and megagenomic analysis of childhood *Mycoplasma pneumoniae* post COVID-19 pandemic: A 6-year study in southern China. Emerg. Microbes Infect..

[4] Yan C., Tong S., Wu Y., Chen Y., Jia X., Guo Y., Cui M., Pei G., Zhang Z., Zhou H. (2025). Macrolide-resistant *Mycoplasma pneumoniae* resurgence in Chinese children in 2023: A longitudinal, cross-sectional, genomic epidemiology study. Lancet Microbe.

[5] Bolluyt D.C., Euser S.M., Souverein D., van Rossum A.M., Kalpoe J., van Westreenen M., Goeijenbier M., Snijders D., Eggink D., Jongenotter F. (2024). Increased incidence of *Mycoplasma pneumoniae* infections and hospital admissions in the Netherlands, November to December 2023. Eurosurveillance.

[6] Waldeck F., Kramer T.S., Boutin S., Matten J., Kramer J., Rupp J. (2025). Re-emergence of *Mycoplasma pneumoniae* before and after COVID-19 pandemic in Germany. BMC Infect. Dis..

[7] Yun K.W. (2025). Growing threat of macrolide-resistant *Mycoplasma pneumoniae* among children: What we know and what we need. J. Korean Med. Sci..

[8] Zhang M., Wen W., Ma Y., Hua Y., Cao Q., Lai H., Ye G., Wu L., Chen G., Ke Q. (2025). Multicenter analysis of the *Mycoplasma pneumoniae* resurgence in China 2023–2024. Sci. Rep..

[9] Huang C., Fu Y. (2025). Feilike mixture targeting the stat1-nlrp3 axis represses the pyroptosis of epithelial cells to alleviate *Mycoplasma pneumoniae* pneumonia. Microb. Pathog..

[10] Zhu M., Lu Y., Wei Y., Hong F., Ji J., Song L. (2025). The nlrp3 inflammasome activation boosts lung injury, inflammation, and macrolide resistance in *Mycoplasma pneumoniae* pneumonia. Cytokine.

[11] Tamura A., Matsubara K., Tanaka T., Nigami H., Yura K., Fukaya T. (2008). Methylprednisolone pulse therapy for refractory *Mycoplasma pneumoniae* pneumonia in children. J. Infect..

[12] You S.Y., Jwa H.J., Yang E.A., Kil H.R., Lee J.H. (2014). Effects of methylprednisolone pulse therapy on refractory *Mycoplasma pneumoniae* pneumonia in children. Allergy Asthma Immunol. Res..

[13] Xiang H., Zheng C., Wang Y., Sun Y., Li C., Cai M., Zhang Y. (2026). Host immune dysfunction and *Mycoplasma pneumoniae* immune evasion: Dual drivers of pathogenesis in *Mycoplasma pneumoniae* pneumonia. Inflamm. Res..

[14] Lai J.F., Zindl C.L., Duffy L.B., Atkinson T.P., Jung Y.W., van Rooijen N., Waites K.B., Krause D.C., Chaplin D.D. (2010). Critical role of macrophages and their activation via myd88-nfκb signaling in lung innate immunity to *Mycoplasma pneumoniae*. PLoS ONE.

[15] Zhao Y., Ma G., Yang X. (2019). Hdac5 promotes *Mycoplasma pneumoniae*-induced inflammation in macrophages through nf-κb activation. Life Sci..

[16] Shen X., Jin Z., Chen X., Wang Z., Yi L., Ou Y., Gong L., Zhu C., Xu G., Wang Y. (2024). Single-cell transcriptome atlas revealed bronchoalveolar immune features related to disease severity in pediatric *Mycoplasma pneumoniae* pneumonia. MedComm.

[17] Li L., Guo R., Zou Y., Wang X., Wang Y., Zhang S., Wang H., Jin X., Zhang N. (2024). Construction and validation of a nomogram model to predict the severity of *Mycoplasma pneumoniae* pneumonia in children. J. Inflamm. Res..

[18] Bodhankar S., Sun X., Woolard M.D., Simecka J.W. (2010). Interferon gamma and interleukin 4 have contrasting effects on immunopathology and the development of protective adaptive immunity against mycoplasma respiratory disease. J. Infect. Dis..

[19] Bodhankar S., Woolard M.D., Sun X., Simecka J.W. (2009). Nk cells interfere with the generation of resistance against mycoplasma respiratory infection following nasal-pulmonary immunization. J. Immunol..

[20] Takahashi R., Shiohara T., Mizukawa Y. (2021). Monocyte-independent and -dependent regulation of regulatory t-cell development in mycoplasma infection. J. Infect. Dis..

[21] Guo H., He Z., Li M., Wang T., Zhang L. (2016). Imbalance of peripheral blood th17 and treg responses in children with refractory *Mycoplasma pneumoniae* pneumonia. J. Infect. Chemother..

[22] Xie S., Wu M., Shang Y., Tuo W., Wang J., Cai Q., Yuan C., Yao C., Xiang Y. (2025). Development and validation of an early diagnosis model for severe mycoplasma pneumonia in children based on interpretable machine learning. Respir. Res..

[23] Ma M., Shi J., Wang W., Huang H., Li X. (2025). Lipidomics changes in bronchoalveolar lavage fluid of refractory *Mycoplasma pneumoniae* pneumonia: Lc-ms-based analysis of potential biomarkers and pathogenesis. Front. Pediatr..

[24] Wang S., Liu C., Ding R., Wang S., Ye Y., He M. (2024). Alterations in gut microbiota and serum metabolites in children with *Mycoplasma pneumoniae* pneumonia. Infect. Drug Resist..

[25] Gao M., Wang K., Yang M., Meng F., Lu R., Zhuang H., Cheng G., Wang X. (2018). Transcriptome analysis of bronchoalveolar lavage fluid from children with *Mycoplasma pneumoniae* pneumonia reveals natural killer and t cell-proliferation responses. Front. Immunol..

[26] Huang X., Gu H., Wu R., Chen L., Lv T., Jiang X., Li H., Guo B., Liu J., Li D. (2024). Chest imaging classification in *Mycoplasma pneumoniae* pneumonia is associated with its clinical features and outcomes. Respir. Med..

[27] Wei W.F., Chu Y.T., Liu Y., Huo J.H., Wang W.M. (2017). Serum metabonomics in mice infected with *Mycoplasma pneumoniae* by uplc-q-tof-ms. Zhongguo Zhong Yao Za Zhi.

[28] Zhang Y., Feng J., Diao J., Gou J., Liang Y., Wang X., Song Z., Zhang B. (2026). Modified ganlu xiaodu decoction attenuates airway barrier injury in *Mycoplasma pneumoniae* pneumonia by inhibiting zbp1-mediated panoptosis. Phytomedicine.

[29] Yang Y., Yi X., Liu C., Zeng Q., Li X., Luo H., Yan P., Gu S., Li C., Xiao L. (2025). Targeting the stat3/acly axis attenuates pulmonary inflammation but delays *mycoplasma pneumoniae* clearance via citrate metabolism. Med. Microbiol. Immunol..

[30] Li G., Wei Y., Wang W., Hu Z., Yang Q., He L., Gao Y., Lai X. (2026). Multi-omics and machine learning-based profiling of severity signatures in *Mycoplasma pneumoniae* infection in children. iScience.

[31] Yang Y., Lian S., Li X., Tang Y., Su Y., Zhang Z., Li M., Guo Y., He Z., Shen Y. (2026). Unveiling metagenomic and metabolomic signatures in mild and severe pneumonia caused by *Mycoplasma pneumoniae* in children. Microb. Genom..

[32] Huang X., Luo Y., Wang J., Zhang X., Chen L., Wu R., Xue Z., Gu H., Li D., Tang H. (2024). Integrative study of pulmonary microbiome, transcriptome and clinical outcomes in *Mycoplasma pneumoniae* pneumonia. Respir. Res..

[33] Jiang Y., Bao C., Zhao X., Chen Y., Song Y., Xiao Z. (2022). Intestinal bacteria flora changes in patients with *Mycoplasma pneumoniae* pneumonia with or without wheezing. Sci. Rep..

[34] Xu Y., Dai Y.L., Lei H., Yuan Y.L., Hu T.L., Wei X., Lan Y.M., Guo L.M. (2026). A study on the correlation between intestinal flora characteristics and serum zinc and iron levels in paediatric patients with *Mycoplasma pneumoniae* pneumonia. BMC Pediatr..

[35] Ubags N.D.J., Marsland B.J. (2017). Mechanistic insight into the function of the microbiome in lung diseases. Eur. Respir. J..

[36] Zaiss Mario M., Rapin A., Lebon L., Dubey Lalit K., Mosconi I., Sarter K., Piersigilli A., Menin L., Walker Alan W., Rougemont J. (2015). The intestinal microbiota contributes to the ability of helminths to modulate allergic inflammation. Immunity.

[37] Feng X., Li L., Yan L., Yan Z., Xu Z., Fan Y., Madjirebaye P., Wu X. (2025). Probiotics attenuate food allergy via short-chain fatty acids-mediated immune modulation and gut barrier restoration. Foods.

[38] Liu Q., Tian X., Maruyama D., Arjomandi M., Prakash A. (2021). Lung immune tone via gut-lung axis: Gut-derived lps and short-chain fatty acids’ immunometabolic regulation of lung il-1β, ffar2, and ffar3 expression. Am. J. Physiol.-Lung Cell. Mol. Physiol..

[39] Li B., Liu M., Du W., Wang S., Xu Z., Zhang X., Zhang Y., Hua S. (2025). Cecropin ad ameliorates pneumonia and intestinal injury in mice with *Mycoplasma pneumoniae* by mediating gut microbiota. BMC Vet. Res..

[40] Calus D., Maes D., Vranckx K., Villareal I., Pasmans F., Haesebrouck F. (2010). Validation of atp luminometry for rapid and accurate titration of mycoplasma hyopneumoniae in friis medium and a comparison with the color changing units assay. J. Microbiol. Methods.

[41] Yacoub E., Chniba I., Khadraoui N., Mlik B., Ben Abdelmoumen Mardassi B. (2023). Antigen-capture enzyme-linked immunosorbent assay for specific detection of *Mycoplasma pneumoniae*. J. Vis. Exp..

[42] Wubbel L., Jafri H.S., Olsen K., Shelton S., Rogers B.B., Gambill G., Patel P., Keyser E., Cassell G., McCracken G.H. (1998). *Mycoplasma pneumoniae* pneumonia in a mouse model. J. Infect. Dis..

[43] Zhang Z., Dou H., Tu P., Shi D., Wei R., Wan R., Jia C., Ning L., Wang D., Li J. (2022). Serum cytokine profiling reveals different immune response patterns during general and severe *Mycoplasma pneumoniae* pneumonia. Front. Immunol..

[44] Shi S., Zhang X., Zhou Y., Tang H., Zhao D., Liu F. (2019). Immunosuppression reduces lung injury caused by *Mycoplasma pneumoniae* infection. Sci. Rep..

[45] He J., Tian W., Feng P., Du T., Long H., He G., Wei H., Zhu X., Jiang X., Zhu C. (2026). Nasal delivery of lacticaseibacillus rhamnosus gg modulates respiratory immune responses and attenuates *Mycoplasma pneumoniae* pneumonia in a murine model. Microbiol. Spectr..

[46] Wu Y., Zhu Q., Liu J., Chuan H., He L., Wang M., Xu L., Zhang R., Liu Y., Liao G. (2025). Influenza a virus and *Mycoplasma pneumoniae* coinfection mediates immune dysregulation and exacerbates disease severity. Int. J. Mol. Sci..

[47] Zhong H., Zeng Z., Gu H., Dong X. (2025). Effect of macrolide resistance and *Mycoplasma pneumoniae* DNA load in bronchoalveolar lavage fluid on immune and inflammatory responses in children with *Mycoplasma pneumoniae* pneumonia. Indian Pediatr..

[48] Xu W., Yang H., Liu H., Tang X., Xu H., Li H., Zhao S. (2020). Bronchoalveolar lavage t cell cytokine profiles and their association with lung function in children with *Mycoplasma pneumoniae*-associated bronchiolitis obliterans. Pediatr. Pulmonol..

[49] Lee Y.C., Chang C.H., Lee W.J., Liu T.Y., Tsai C.M., Tsai T.A., Tsai C.K., Kuo K.C., Chen C.C., Niu C.K. (2021). Altered chemokine profile in refractory *Mycoplasma pneumoniae* pneumonia infected children. J. Microbiol. Immunol. Infect..

[50] Schaunaman N., Sanchez A., Dimasuay K.G., Pavelka N., Numata M., Alam R., Martin R.J., Chu H.W. (2019). Interleukin 1 receptor-like 1 (il1rl1) promotes airway bacterial and viral infection and inflammation. Infect. Immun..

[51] Takashima T., Zeng C., Murakami E., Fujiwara N., Kohara M., Nagata H., Feng Z., Sugai A., Harada Y., Ichijo R. (2025). Involvement of lncrna mir205hg in idiopathic pulmonary fibrosis and il-33 regulation via alu elements. JCI Insight.

[52] Hao Y.-P. (2024). Evaluating the role of interleukin-2 and interleukin-12 in pediatric patients with concurrent *Mycoplasma pneumoniae* and epstein-barr virus infections. World J. Clin. Cases.

[53] Zhu Y., Luo Y., Li L., Jiang X., Du Y., Wang J., Li H., Gu H., Li D., Tang H. (2023). Immune response plays a role in *Mycoplasma pneumoniae* pneumonia. Front. Immunol..

[54] Zhu N., Cai C., Zhou A., Zhao X., Xiang Y., Zeng C. (2017). Schisandrin b prevents hind limb from ischemia-reperfusion-induced oxidative stress and inflammation via MAPK/NF-κb pathways in rats. BioMed Res. Int..

[55] Hong H., Zeng Y., Jian W., Li L., Lin L., Mo Y., Liu M., Fang S., Xia Y. (2018). Cdk7 inhibition suppresses rheumatoid arthritis inflammation via blockage of NF-κb activation and il-1β/il-6 secretion. J. Cell. Mol. Med..

[56] Li Z., Hao C., Jia G., Liang Q., Wang Q., Wu Y., Tang Y., Ji W., Shen Y., Wang F. (2025). Multi-omics analysis of host airway responses in pediatric *Mycoplasma pneumoniae* pneumonia reveals potential mechanisms of disease exacerbation caused by co-infection. npj Biofilms Microbiomes.

[57] Wang B., Wu L., Chen J., Dong L., Chen C., Wen Z., Hu J., Fleming I., Wang D.W. (2021). Metabolism pathways of arachidonic acids: Mechanisms and potential therapeutic targets. Signal Transduct. Target. Ther..

[58] Polito A., Aboab J., Annane D. (2006). Adrenal insufficiency in sepsis. Rev. Bras. Ter. Intensiv..

[59] Peeters B., Langouche L., Van den Berghe G. (2017). Adrenocortical stress response during the course of critical illness. Compr. Physiol..

[60] Adak A., Khan M.R. (2019). An insight into gut microbiota and its functionalities. Cell. Mol. Life Sci..

[61] de Vos W.M., Tilg H., Van Hul M., Cani P.D. (2022). Gut microbiome and health: Mechanistic insights. Gut.

[62] Shi D.W., Wang D.M., Ning L.H., Li J., Dong Y., Zhang Z.K., Dou H.W., Wan R.J., Jia C.M., Xin L. (2022). Using 16s rdna sequencing technology to preliminarily analyze intestinal flora in children with *Mycoplasma pneumoniae* pneumonia. Biomed. Environ. Sci..

[63] Unrug-Bielawska K., Sandowska-Markiewicz Z., Piątkowska M., Czarnowski P., Goryca K., Zeber-Lubecka N., Dąbrowska M., Kaniuga E., Cybulska-Lubak M., Bałabas A. (2025). Comparative analysis of gut microbiota responses to new sn-38 derivatives, irinotecan, and folfox in mice bearing colorectal cancer patient-derived xenografts. Cancers.

[64] von Knethen A., Neb H., Morbitzer V., Schmidt M.V., Kuhn A.M., Kuchler L., Brüne B. (2011). PPARγ stabilizes HO-1 mRNA in monocytes/macrophages which affects IFN-β expression. Free Radic. Biol. Med..

