# Supplementary material for: Multi-Omics Profiling Identifies Immune–Metabolic Signatures and Gut Microbial Biomarkers in a Murine Model of Mycoplasma pneumoniae Pneumonia
Source: Biology (Basel). 2026 Aug 31;15(17):1469. doi: 10.3390/biology15171469 (PMC13564507; doi:10.3390/biology15171469)
Supplement: Supplementary file 1 [file biology-15-01469-s001.zip › biology-4491414-supplementary.pdf]

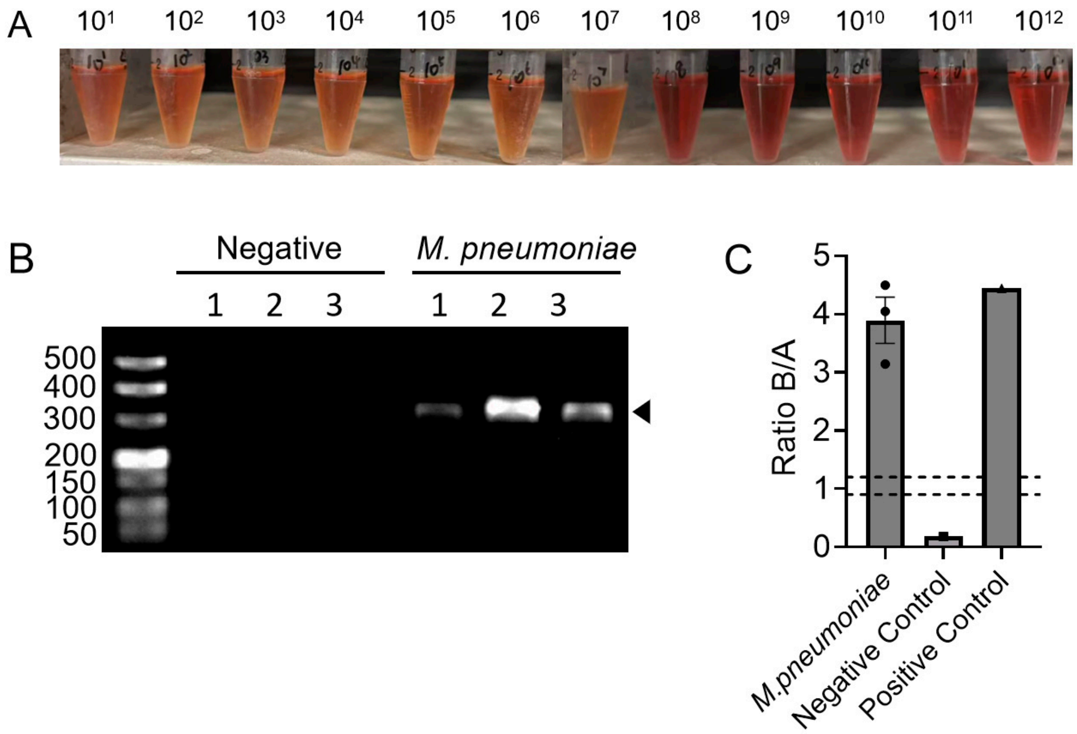

Supplemental Figure S1: Determination of MP concentration and identification. A CCU titer determination of MP by color change method, suggesting MP concentration was determined to be 10^7CCU/mL.B PCR identification of MP in lung tissues. C ELISA identification of MP antigen in lung tissue lysates. Data are presented as mean ± SD.

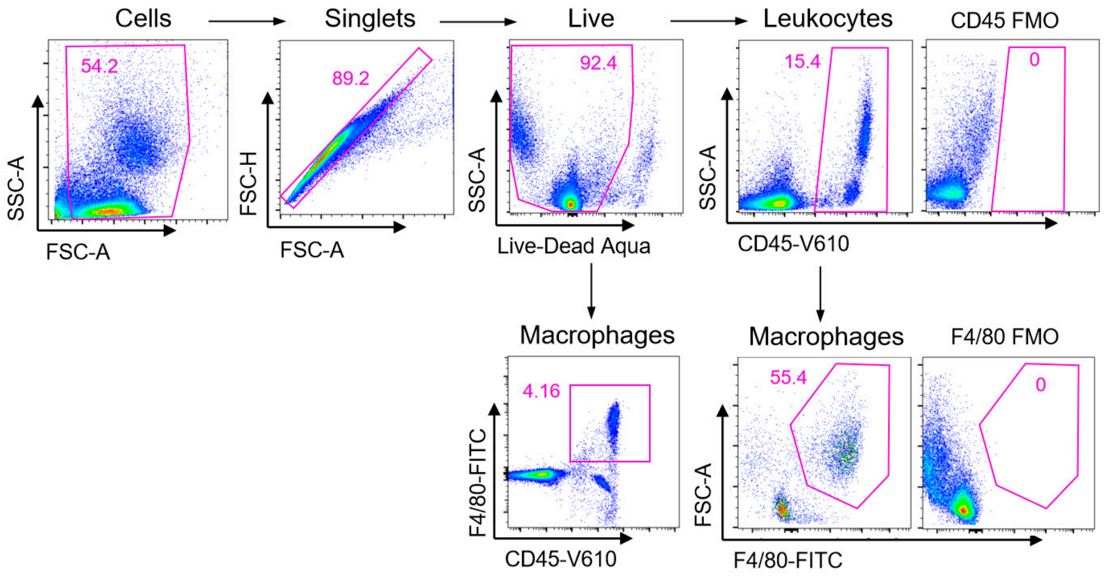

Supplemental Figure S2: Gating strategy for alveolar macrophage identification in BALF. Cells were first gated on forward scatter area (FSC-A) versus side scatter area (SSC-A) to exclude debris, followed by doublet discrimination using FSC-A versus FSC-H to select singlets. Live cells were then gated as negative for Live-Dead Aqua staining. Among live cells, leukocytes were identified as $CD45^+$ cells, and alveolar macrophages were further defined as $CD45^+F4/80^+$ cells. Fluorescence minus one (FMO) were used to define the positive gates for CD45 and F4/80. Representative plots are shown with the percentages of cells in each gate.

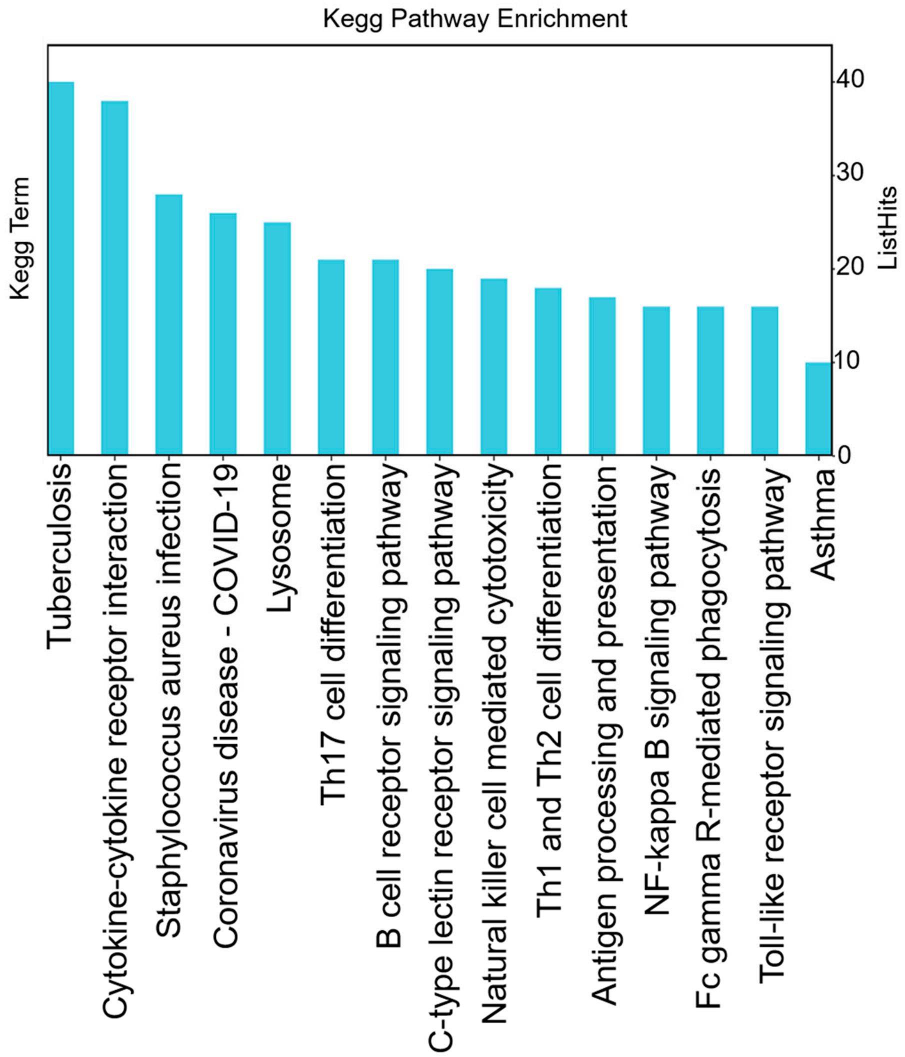

Supplemental Figure S3: Transcriptomic Profiling Reveals Activation of Anti-Infective Immune and Inflammatory in MP-Infected Lung. (A) KEGG pathway enrichment analysis of differentially expressed genes in lung tissues upon *Mycoplasma pneumoniae* infection.

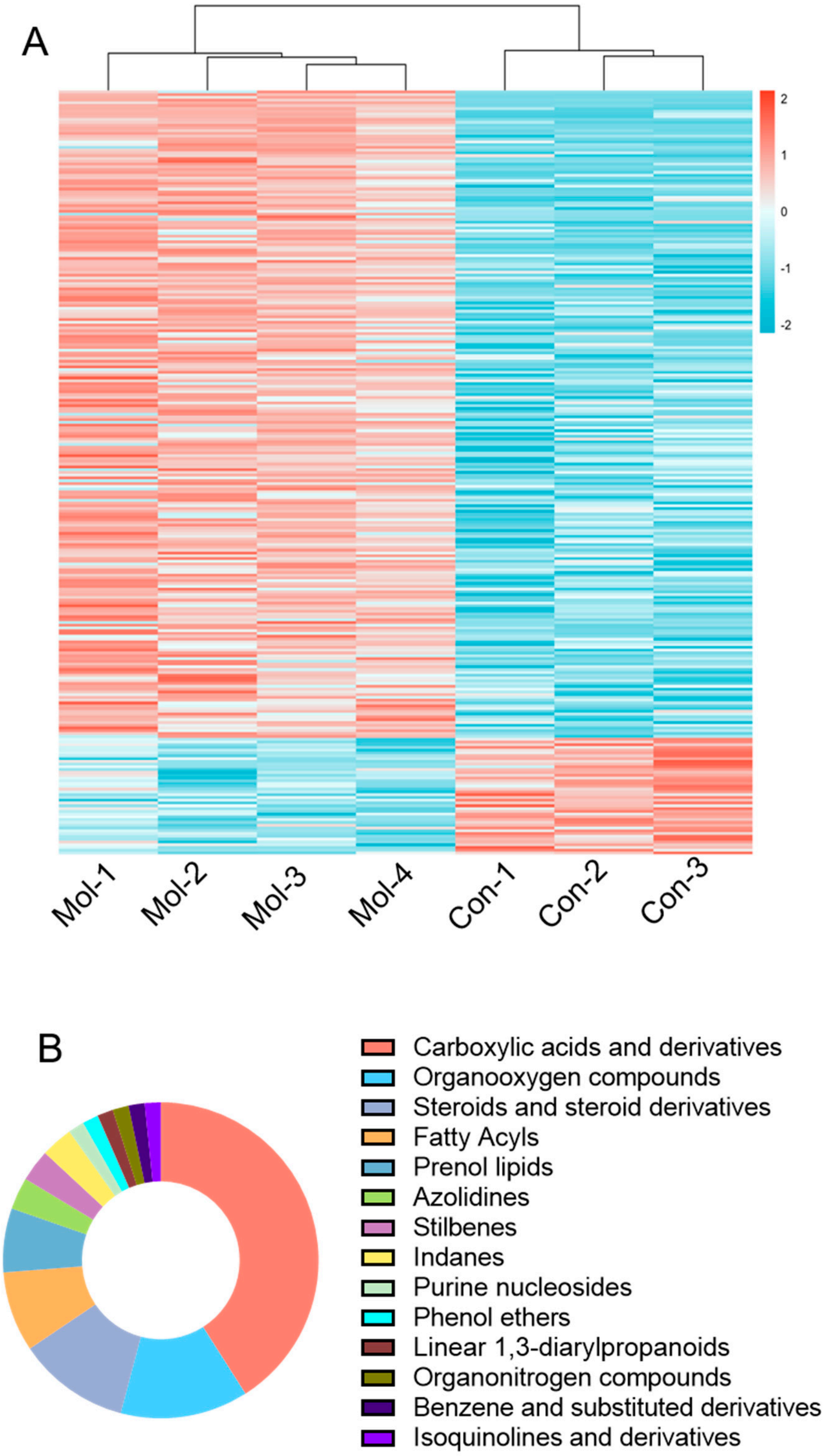

Supplemental Figure S4: Dysbiosis of Colonic Microbiota and Fecal Metabolic Disruption Correlate with Inflammation upon MP Infection. (A) Hierarchical clustering heatmap of fecal differential metabolites in Con and Mol group. (B) Classify the differential metabolites of feces according to Human Metabolome Database.

Table S1 Primer sequence used for mRNA RT-qPCR

Gene	Sequences (5'→3')
Tnf-α	Forward: TAGCCCACGTCGTAGCAAAC<br>Reverse: TGTCTTTGAGATCCATGCCGT
Il-1β	Forward: TGCCACCTTTTGACAGTGATG<br>Reverse:TGATGTGCTGCTGCGAGATT
β-actin	Forward: CGTCAGGCAGCTCATAGCTCTTCT<br>Reverse:TGGCTACAGCTTCACCACCACAG

Table S2 Histopathological scoring system for lung tissues.

Parameter	0 points	1 point	2 points	3 points
Peribronchial/bronchiolar infiltrates (extent)	None	Few (<25%)	Many (25–75%)	Most (>75%)
Peribronchial/bronchiolar infiltrates (quality)	None, occasional mild infiltration or peribronchial lymphoid aggregates	Mild, abnormal, often with discontinuous rings	Moderate, complete or crescent-shaped ring, <5 cells thick	Severe, complete ring, >5-10 cells thick
Bronchiolar/bronchial luminal exudate	None	Mild (<25% luminal closure)	Severe (>25% luminal closure)	—
Perivascular infiltrates	None	Few (<10%)	Many (10–50%)	Most (>50%)
Parenchymal pneumonia	None	Mild, patchy parenchymal infiltration	Severe, confluent parenchymal infiltration	—

Table S3 The number and percent of metabolites in class

Class	Number	Percent
Fatty Acyls	158	23.76%
Carboxylic acids and derivatives	118	17.74%
Glycerophospholipids	87	13.08%
Organooxygen compounds	74	11.13%
Prenol lipids	60	9.02%
Steroids and steroid derivatives	53	7.97%
Benzene and substituted derivatives	29	4.36%
Organonitrogen compounds	25	3.76%
Indoles and derivatives	12	1.80%
Purine nucleotides	10	1.50%
Purine nucleosides	8	1.20%
Hydroxy acids and derivatives	8	1.20%
Imidazopyrimidines	8	1.20%
Isoflavonoids	8	1.20%
Flavonoids	7	1.05%
